# Prognostic Values of Vimentin Expression and Its Clinicopathological Significance in Non-Small Cell Lung Cancer: A Meta-Analysis of Observational Studies with 4118 Cases

**DOI:** 10.1371/journal.pone.0163162

**Published:** 2016-09-22

**Authors:** Zhihua Ye, Xin Zhang, Yihuan Luo, Shikang Li, Lanshan Huang, Zuyun Li, Ping Li, Gang Chen

**Affiliations:** 1 Department of Pathology, First Affiliated Hospital of Guangxi Medical University, No.6 Shuangyong Road, Nanning, Guangxi Zhuang Autonomous Region 530021, P. R. China; 2 Department of Thoracic and Cardiovascular Surgery, First Affiliated Hospital of Guangxi Medical University, No.6 Shuangyong Road, Nanning, Guangxi Zhuang Autonomous Region 530021, P. R. China; University of South Alabama Mitchell Cancer Institute, UNITED STATES

## Abstract

**Background:**

Vimentin is a member of the intermediate filament proteins and a canonical marker of the epithelial-mesenchymal transition (EMT), which is pivotal in tumorigenesis, metastasis and invasion in non-small cell lung cancer (NSCLC). The current meta-analysis aimed to investigate the associations between vimentin and prognosis and progression in NSCLC.

**Methods:**

Databases with literature published in English, including PubMed, Web of Science, Embase, Science Direct, Wiley Online Library, Ovid, Cochrane Central Register of Controlled Trials, LILACS and Google Scholar, and the CNKI, VIP, CBM and WanFang databases in Chinese were used for the literature search. The key terms included (1) ‘vimentin’ OR ‘vim’ OR ‘vmt’ OR ‘vm’ OR ‘hel113’ OR ‘ctrct30’ and (2) ‘pulmon*’ OR ‘lung’ OR ‘alveolar’ and (3) ‘cancer’ OR ‘carcinoma’ OR ‘tumor’ OR ‘adenocarcinoma’ OR ‘squamous’ OR ‘neoplas*’ OR ‘malignan*’. The data were combined by random effect model and the H value and I^2^ were used to assess the heterogeneity. All the meta-analysis was conducted using Stata 12.0.

**Results:**

Thirty-two qualified studies (4118 cases) were included in the current meta-analysis. Twelve studies with 1750 patients were included to assess the significance of vimentin in the overall survival (OS) of NSCLC; the pooled hazard ratio (HR) was 1.831 (confidence interval (CI): 1.315–2.550, P<0.001) in the univariate analysis and 1.266 (CI: 0.906–1.768, P = 0.167) in the multivariate analysis. Four studies with 988 cases were applicable to determine the significance of vimentin in the disease-free survival (DFS) of NSCLC; the pooled HR of the DFS was 1.224 (CI: 0.921–1.628, P = 0.164) in the univariate analysis and 1.254 (CI: 0.985–1.956, P = 0.067) in the multivariate analysis. Regarding the relationships between vimentin and clinicopathological factors, the pooled odds ratio (OR) with 3406 NSCLCs indicated that up-regulated vimentin was associated with smoking (OR = 1.359, CI: 1.098–1.683, P = 0.004), poor differentiation (OR = 2.133, CI: 1.664–2.735, P<0.001), an advanced TNM stage (OR = 3.275, CI: 1.987–5.397, P<0.001), vascular invasion (OR = 3.492, CI: 1.063–11.472, P = 0.039), lymph node metastasis (OR = 2.628, CI: 1.857–3.718, P<0.001), recurrence (OR = 1.631, CI: 1.052–2.528, P = 0.029) and pleural invasion (OR = 2.346, CI: 1.397–3.941, P = 0.001). There was no significant correlation between vimentin and age, gender, diameter, T stage, distant metastasis, or marginal invasion (P>0.05).

**Conclusion:**

An overexpression of vimentin may predict the progression and an unfavorable survival of NSCLC. Vimentin may represent a helpful biomarker and a potential target for the treatment strategies of NSCLC. Additional, prospective studies with large samples are necessary to confirm the significance of vimentin in NSCLC.

## Introduction

Lung cancer is considered the most frequent type of cancer and is regarded as the leading cause of cancer-related deaths worldwide [1]. In China, a similar trend is present in which the incidence and mortality of lung cancer has rapidly increased, and lung cancer currently ranks as the first type of dominating malignancies [2]. Non-small cell lung cancer (NSCLC), which comprises approximately 85%, is the predominant type of lung cancer. As a result of a deficiency in efficacious biomarkers for early diagnosis, the majority of NSCLC sufferers are diagnosed in an advanced stage [3, 4]. There is increasing evidence for therapeutic targets, such as EGFR, HER2, ALK, ROS1, BRAF, MET, VEGF, and FGFR1, which have received attention in clinical research. However, the prognosis for patients with NSCLC remains poor[5–7]. Thus, highly sensitive and specific biomarkers for the prediction of progression and prognosis are urgently demanded to improve the survival of patients with lung cancer.

Vimentin is a highly conserved intermediate filament protein with 57 KDa and is a member of the cytoskeletal proteins; it is observed in various cell types[8]. As an important marker of the EMT, vimentin is essential to the progression and prognosis of cancer through the EMT and the corresponding signaling pathways, which contribute to the tumorigenesis, metastasis, invasion and drug resistance of various cancers[9, 10]. Accumulating evidence indicates that vimentin is critical for the progression and prognosis of lung cancer [11–13]. However, according to published studies, the role of vimentin is inconsistent, and the function of vimentin remains controversial regarding whether it predicts a better or worse prognosis. Thus, the current meta-analysis aimed to determine the role of vimentin expression in the progression and prognosis of NSCLC.

## Methods

### Publication search

In this meta-analysis, databases with literature published in English, including PubMed, Web of Science, EMBASE, Science Direct, Wiley Online Library, Ovid, Cochrane Central Register of Controlled Trials, LILACS and Google Scholar, and the CNKI, VIP, CBM and WanFang databases in Chinese were used for the literature search. The studies qualified for the present meta-analysis were updated on October 31, 2015. The search terms were as follows: ‘vimentin’ OR ‘vim’ OR ‘vmt’ OR ‘vm’ OR ‘hel113’ OR ‘ctrct30’ and ‘pulmon*’ OR ‘lung’ OR ‘alveolar’ and ‘cancer’ OR ‘carcinoma’ OR ‘tumor’ OR ‘adenocarcinoma’ OR ‘squamous’ OR ‘neoplas*’ OR ‘malignan*’.

### Selection criteria

The literature was screened according to the following criteria. The inclusion criteria for the primary studies included 1) patients with a diagnosis of primary or metastatic lung cancer confirmed by pathology, 2) the determination of vimentin protein or vimentin mRNA in the tissues of NSCLC patients using immunohistochemistry (IHC) or real-time reverse transcription-polymerase chain reaction (qRT-PCR), 3) investigation of the relation between vimentin expression and the overall survival (OS), disease free survival (DFS) or clinicopathological features (age, gender, tumor size, distant metastasis, subtype, grading, TNM stage, or lymph node metastasis),and the survival data may be directly or indirectly obtained, 4) publication in English or Chinese, and 5) when authors had several publications or reported data on the same patient population, only the most recent or largest sample study was included. The exclusion criteria for the primary studies included 1) reviews, letters, conference data, and case reports, 2) an overlap among articles or duplicate data, 3) the use of animals or cell lines, 4) insufficient data availability for the clinicopathological features, estimation of the HR or the 95% CI, 5) vimentin was combined with other markers or a positive EMT to predict the prognosis and progression in NSCLC, or 6) a poor study quality.

### Data extraction

Two authors were responsible for independent data collection; when the two authors had inconsistent opinions regarding a study, a third author made the final decision. Data regarding the names of the first authors, year of publication, countries, numbers of patients, technology of detection and clinicopathological parameters, including age, gender, diameter, histology, smoking status, differentiation, T stage, TNM stages, vascular invasion, lymph node metastasis, distant metastasis, recurrence and marginal invasion, were extracted to calculate pooled ORs. Furthermore, the survival analysis type and HR with 95% CI were collected.

### Quality assessment

Two authors (Zhihua Ye and Xin Zhang) independently evaluated the quality of the eligible studies using the Newcastle-Ottawa Scale (NOS)[14]. In the NOS, each study is assessed using three points: the selection of cohorts, the comparability of cohorts and the ascertainment of outcomes. Each study was able to be recoded with no more than one star of each item in the Selection and Outcomes section. Moreover, in the Comparability section, a maximum of two stars was recorded. The total stars for each study were calculated by the sum of the three points, which resulted in a maximum of nine stars. A study with more stars indicated a better quality.

### Statistical analysis

All data analysis was conducted using Stata 12.0 and the metan package contributed to the combination of the data. Combined ORs and corresponding 95% CIs were used to evaluate the relationships between vimentin and the clinicopathological factors, including age, gender, tumor size, histology, smoking status, differentiation, T stage, TNM stages, vascular invasion, lymph node metastasis, distant metastasis, recurrence and marginal invasion. The prognostic significance of vimentin in the patients with lung cancer was appraised by pooled HRs with 95% CIs. The HRs and 95% CIs were determined directly from the univariate or multivariate survival analysis and indirectly from Kaplan–Meier survival curves as reported by Parmar[15]. The H value and its 95%CI was used to estimate the heterogeneity among the eligible studies[16–18]. Moreover, Q test (chi-squared test) and I^2^ statistic with its 95%CI were also reported[19]. Random-effects (RE) models were performed regarding of the heterogeneity since RE models are more conservative and can provide better estimates with wider confidence intervals[20, 21]. Furthermore, we conducted Begg’s test to assess the publication bias, and it was considered statistically significant with a P value less than 0.05.

## Results

### Study characteristics

**Fig 1:**
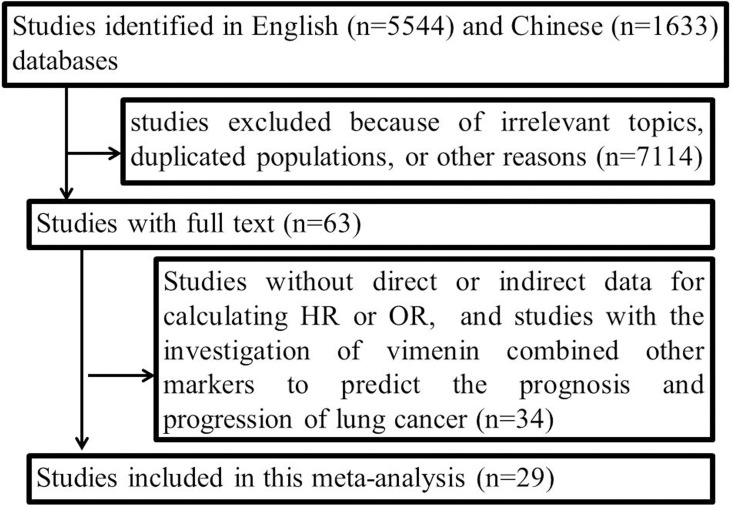
Flow chart of literature search.

The flow chart of the literature search is shown in Fig 1. Thirty-two studies with 4118 cases were included with a closing date of October 31, 2015. Six studies (822 cases) only investigated the prognosis[11, 22–26], and eighteen studies (1946 cases) only assessed the correlation between vimentin and progression in NSCLC[13, 27–43]. Moreover, eight studies (1350 cases) provided data regarding both prognosis and progression in NSCLC[12, 44–50]. The detailed information of the studies is presented in Tables 1 and 2.

**Table 1:** Characteristics of the included studies for the overall survival (OS) analysis.

First author	Year	Country	Cancer	Stage	Follow up(M)	Case(N)	Treatment	Technique	Location of staining	Cut off	Overall Survival		NOS
											Univariate analysis	Multivariate analysis	
											HR (95% CI)	HR (95% CI)	
Che JJ	2015	China	SCC	I, IIIa	70	103	pulmonectomy	IHC	membrane	>0%	2.08(1.34–3.21)	1.918(1.107–3.324)	8
Hroyasu N	2015	Japan	AC	I- III	38.1*	101	pulmonectomy	IHC	Cytoplasm	>median score		2.720 (1.23–6.52)	8
Terumasa S	2015	Japan	AC	Ia-IIIb	63*	239	pulmonectomy	IHC	Cytoplasm	scores≥2	2.41(1.3–4.46)		8
Xu MM	2014	China	NSCLC	I- III	60	150	pulmonectomy	IHC	Cytoplasm	>20%		0.580(0.389–0.867)	8
Kong FF	2014	China	NSCLC	I- IV	60	68	pulmonectomy	IHC	Cytoplasm	scores≥1	2.596(1.386–4.864)	1.287(0.642–2.580)	8
Zhang X	2014	China	NSCLC	I- IIIa	60	118	pulmonectomy	IHC	Cytoplasm	score≥3		1.664(1.031–2.686)	8
Guan XQ	2014	China	NSCLC	I-IV	60	500	pulmonectomy	IHC	Cytoplasm	score≥3	0.842(0.626–1.132)		7
Chao Z	2013	China	NSCLC	I- IV	39*	119	pulmonectomy	IHC	Cytoplasm	≥10%	2.21(1.21–4.039)	1.012(0.52–1.968)	8
Zhang H	2013	China	SCC	I- IIIa	51.5*	204	pulmonectomy	IHC	Cytoplasm	score≥4	1.435(1.086–1.896)	1.17(0.877–1.561)	8
Frank R (a)	2012	America	NSCLC	Ia- IV	24	57	chemotherapy	IHC	Cytoplasm	≥10%		0.65(0.31–1.38)	8
Frank R (b)	2012	America	NSCLC	Ia- IV	24	38	placebo	IHC	Cytoplasm	≥10%		2.32(1.09–4.94)	8
Zhou H	2012	China	NSCLC	I- III	41.5*	52	pulmonectomy	IHC	Cytoplasm	score≥3	3.45(1.66–7.11)		7
Shi YL	2011	China	NSCLC	I-IV	54	165	pulmonectomy	IHC	Cytoplasm	score≥4	2.18(1.43–3.24)		7
Ye T	2009	China	NSCLC	IIIa	36	75	pulmonectomy	IHC	Cytoplasm	score≥1	1.79(0.92–3.49)		7

NSCLC: non-small cell lung cancer, SCC: squamous cell carcinoma, AC: adenocarcinoma, IHC: immunohistochemistry, NOS: Newcastle-Ottawwwa-Scale.

*: median follow-up time.

**Table 2:** Characteristics of the included studies for the disease-free survival (DFS) analysis.

First author	Year	Country	Cancer	Stage	Follow up(M)	Case(N)	Treatment	Technique	Location of staining	Cut off	Disease-free Survival		NOS
											Univariate analysis	Multivariate analysis	
											HR (95% CI)	HR (95% CI)	
Hroyasu N	2015	Japan	AC	I- III	38.1*	101	pulmonectomy	IHC	Cytoplasm	>median score		1.72(1.00–2.99)	8
Guan XQ	2014	China	NSCLC	I-IV	60	500	pulmonectomy	IHC	Cytoplasm	score≥3	1.015(0.800–1.287)		8
Zhang H	2013	China	SCC	I- IIIa	51.5*	204	pulmonectomy	IHC	Cytoplasm	score≥4	1.474(1.117–1.944)	1.164(0.873–1.552)	8
Yasuhiro C	2011	Japan	NSCLC	I- III	53.7*	183	pulmonectomy	IHC	NR	≥50%	1.326(0.634–2.770)	1.144(0.535–2.443)	8

NSCLC: non-small cell lung cancer, SCC: squamous cell carcinoma, AC: adenocarcinoma, IHC: immunohistochemistry, NOS: Newcastle-Ottawwwa-Scale.

*: median follow-up time.

### Association of vimentin expression and OS

**Fig 2:**
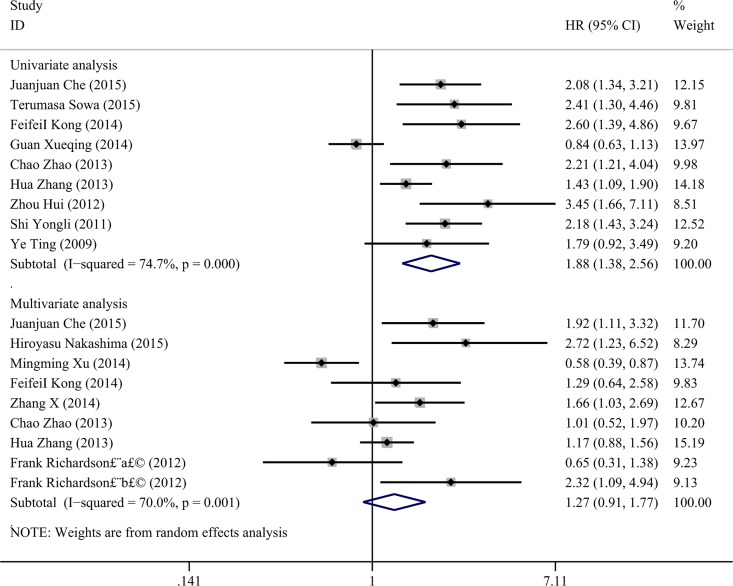
Forest plots of vimentin expression and OS rate in NSCLC via univariate and multivariate analyses.

We initially focused on the relation between vimentin and OS in NSCLC; thirteen qualified studies with a total number of 1750 cases were included to estimate the significance of vimentin in the overall survival of NSCLC. Data for the HR and 95% CI for the overall OS analysis were extracted indirectly from survival curves and directly from univariate and multivariable analyses as described in the methods. The univariate analysis included nine studies with 1525 NSCLCs, and eight studies with 958 NSCLCs were included in the multivariable analysis. In the univariate analysis, a random-effect model was used with the heterogeneity test (P<0.001, I^2^ = 75%, 95%CI: 51%-87%, H = 2.0, 95%CI: 1.4–2.8), and the results of the pooled HR indicated that positive vimentin expression predicted an unfavorable OS (HR = 1.878, CI: 1.378–2.562, P<0.001, Fig 2). However, the pooled HR of the OS in the multivariable analysis was 1.266 with a 95% CI of 0.906 to 1.768 (P = 0.167) using the random-effect model with the heterogeneity test (P = 0.001, I^2^ = 70.0%, 95%CI: 40%-85%, H = 1.8, 95%CI: 1.3–2.6, Fig 2). In addition, an analysis of the subgroups was performed, and the results are shown in Table 3.

**Table 3:** Subgroup analysis of HR in overall survival (OS) by univariate and multivariable analyses.

Group	Study (N)	Heterogeneity test			HR (95% CI)	P
		I^2(^95%CI)	H^(^95%CI)	P		
Overall survival(U)	9	75%(51%-87%)	2.0(1.4–2.8)	<0.001	1.878(1.378–2.562)	<0.001
Country						
China	8	77%(53%-88%)	2.1(1.5–2.9)	<0.001	1.831 (1.315–2.550)	<0.001
Japan	1	-		-	-	-
Sample						
≤200	6	0%(0%-75%)	1.0(1.0–2.0)	0.831	2.251(1.806–2.805)	<0.001
>200	3	83%(49%-94%)	2.4(1.4–4.3)	0.002	1.352(0.807–2.264)	0.252
Cancer type						
SCC	2	55.7%		0.160	1.724(1.060–2.802)	0.028
AC	1	-		-	-	-
Year						
2013–2015	6	80%(57%-91%)	2.2(1.5–3.3)	<0.001	1.736(1.159–2.601)	0.007
2009–2012	3	0.0%(0%-90%)	1.0(1.0–3.1)	0.851	2.229(1.638–3.034)	<0.001
Overall survival(M)	9	70%(40%-85%)	2.0(1.3–2.6)	0.001	1.266(0.906–1.768)	0.167
Region						
China	6	71%(31%-87%)	1.8(1.2–2.8)	0.005	1.168(0.814–1.676)	0.4
Japan and USA	3	75%(18%-92%)	2.0(1.1–3.6)	0.018	1.584(0. 643–3.899)	0.317-
Sample						
≤200	8	74%(47%-87%)	2.0 (1.4–2.8)	<0.001	1.296(0.851–1.975)	0.225
>200	1	-		-	-	-
Cancer type		-		-	-	-
SCC	2	58.9%		0.119	1.414(0.883–2.265)	0.149
AC	1	-			-	-
Year						
2013–2015	7	72%(38%-87%)	1.9(1.3–2.8)	0.002	1.273(0.886–1.830)	0.191
2009–2012	2	81.9%		0.019	1.226(0.352–4.267)	0.748

U: univariate analysis, M: multivariable analysis, SCC: squamous cell carcinoma, AC: Adenocarcinoma.

### Association of vimentin expression and DFS

**Fig 3:**
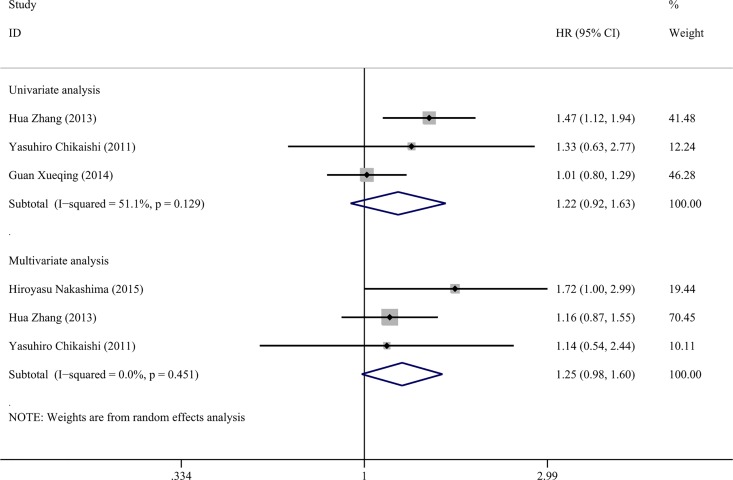
Forest plots of vimentin expression and DFS rate in NSCLC via univariate and multivariate analyses.

Four studies were included in the meta-analysis to assess the significance of vimentin in the DFS of NSCLC. Survival data were extracted from univariate analyses with 3 studies of 887 NSCLC cases included, as well as multivariable analyses with 3 studies of 488 NSCLC cases included. The pooled HR was 1.224 (CI: 0.921–1.628, P = 0.164, Fig 3) in the univariate analysis using the random-effect model with the heterogeneity test (P = 0.129, I^2^ = 51%, 95%CI: 0%-86%, H = 1.4, 95%CI: 1.0–2.7); in the multivariable analysis, the pooled HR was 1.254 (CI: 0.985–1.956, P = 0.067) via the random-effect model based on the heterogeneity test (P = 0.451, I^2^ = 0%, 95%CI: 0%-90%, H = 1.0, 95%CI: 1.0–3.1, Fig 3).

### Correlations between vimentin and clinicopathological characteristics

**Fig 4:**
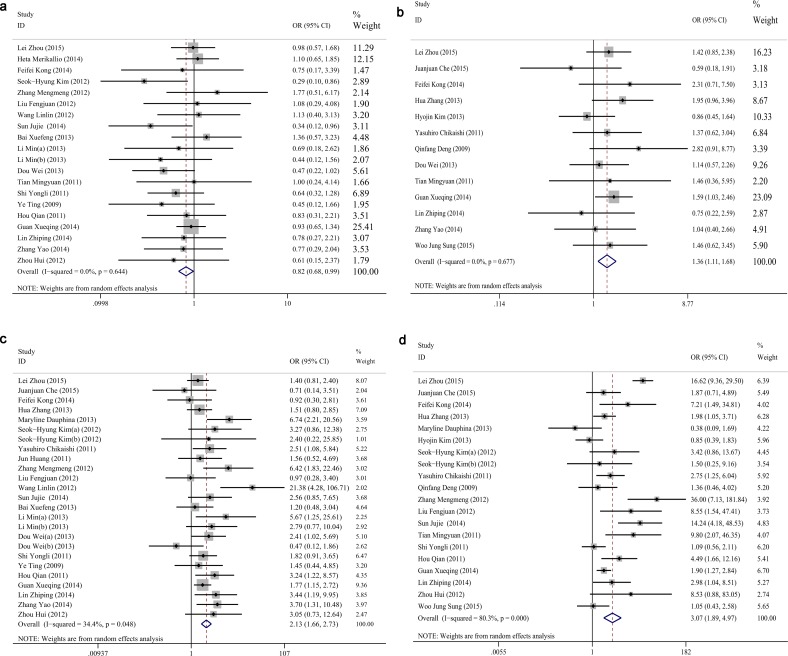
Forest plots of ORs for associations between vimentin and clinicopathological characteristics in NSCLC. (a) Histology, (b) Smoking status, (c) Differentiation, and (d) TNM stages.

**Fig 5:**
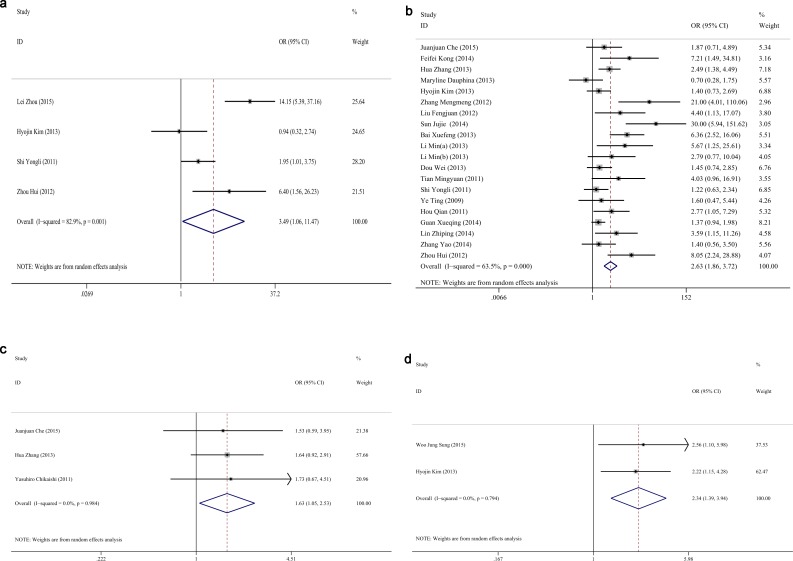
Forest plots of ORs for associations between vimentin and clinicopathological characteristics in NSCLC. (a) Vascular invasion, (b) Lymph node metastasis, (c) Recurrence, and (d) Pleural invasion.

Finally, we analyzed the relationships between vimentin and the clinicopathological characteristics; twenty-six studies were analyzed with a sample size of 3296 NSCLCs. The pooled ORs of vimentin in various parameters are summarized in Table 4. The results of the pooled OR indicated that vimentin positivity was associated with the histology of adenocarcinoma (OR = 0.821, CI: 0.684–0.986, P = 0.034, Fig 4A), smoking (OR = 1.362, CI: 1.105–1.678, P = 0.003, Fig 4B), poor differentiation (OR = 2.133, CI: 1.664–2.735, P<0.001, Fig 4C), advanced TNM stage (OR = 3.275, CI: 1.987–5.397, P<0.001, Fig 4D), vascular invasion (OR = 3.492, CI: 1.063–11.472, P = 0.039, Fig 5A), lymph node metastasis (OR = 2.628, CI: 1.857–3.718, P<0.001, Fig 5B), recurrence (OR = 1.632, CI: 1.054–2.529, P = 0.028, Fig 5C) and pleural invasion (OR = 2.344, CI: 1.395–3.393, P = 0.001, Fig 5D). There was no significant correlation between vimentin and age, gender, diameter, T stage, distant metastasis, or marginal invasion (P>0.05).

**Table 4:** Analysis of relationships between vimentin and clinicopathological variables in NSCLC.

Clinicalpathological Variable	No. of Studies	model	Cases (N)	Pooled OR	95% CI		P value	Test for Heterogeneity		
								P value	I^2(^95%CI)	H^(^95%CI)
Age(≥ 60/<60)	24	Random effect	2810	0.927	0.789	1.088	0.353	0.799	0%(0%-45%)	1.0(1.0–1.3)
Gender(male/female)	25	Random effect	3105	1.122	0.944	1.332	0.192	0.473	0%(0%-44%)	1.0(1.0–1.3)
Diameter(>3.0cm/≤3.0 cm)	11	Random effect	1166	1.317	0.919	1.888	0.133	0.123	34%(0%-68%)	1.2(1.0–1.8)
Histology(SCC/AC)	20	Random effect	2259	0.821	0.684	0.986	0.034	0.644	0%(0%-48%)	1.0(1.0–1.4)
Smoking status(yes/no)	12	Random effect	2050	1.362	1.105	1.678	0.004	0.677	0%(0%-57%)	1.0(1.0–1.5)
Differentiation(poorly/moderate or well)	25	Random effect	2856	2.133	1.664	2.735	<0.001	0.048	34%(0%-60%)	1.2(1.0–1.6)
T stage(T2 or T3/T1)	6	Random effect	957	1.044	0.704	1.548	0.830	0.498	0%(0%-75%)	1.0(1.0–2.0)
TNM stages(III or IV/I or II)	20	Random effect	2661	3.069	1.894	4.974	<0.001	<0.001	80%(70%-87%)	2.3(1.8–2.8)
Vascular invasion(yes/no)	4	Random effect	722	3.492	1.063	11.472	0.039	0.001	83%(56%-93%)	2.4(1.5–3.9)
Lymph node metastasis(yes/no)	20	Random effect	2395	2.628	1.857	3.718	<0.001	<0.001	64%(41%-77%)	1.7(1.3–2.1)
Recurrence(yes/no)	3	Random effect	490	1.632	1.054	2.529	0.028	0.984	0%(0%-490%)	1.0(1.0–3.1)
Distant metastasis	2	Random effect	248	3.975	0.724	21.807	0.112	0.069	70% (-)	(-)
Marginal invasion	2	Random effect	477	2.429	0.789	7.473	0.122	0.025	80% (-)	(-)
Pleural invasion	2	Random effect	290	2.344	1.395	3.393	0.001	0.794	0% (-)	(-)

SCC: squamous cell carcinoma, AC: Adenocarcinoma.

### Publication bias and sensitivity analysis

**Fig 6:**
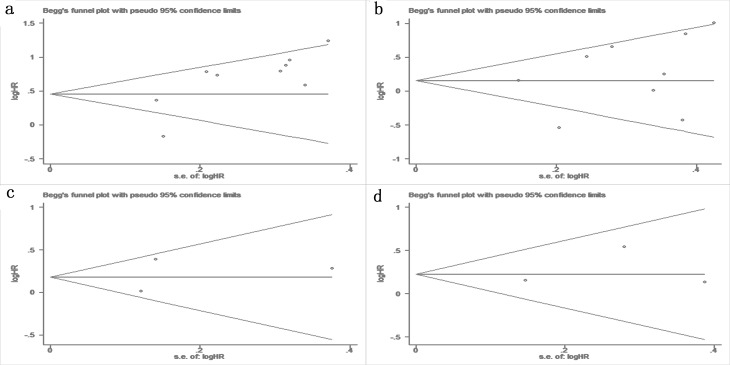
Funnel plots for publication bias. (a) OS in univariate analysis, (b) OS in multivariate analysis, (c) DFS in univariate analysis, and (d) DFS in multivariate analysis.

Begg’s test was used to examine the publication bias of the eligible studies, and no obvious publication bias was identified in the OS (P = 0.175 in univariate analysis, Fig 6A, P = 0.251 in multivariable analysis, Fig 6B) or the DFS (P>0.99 in both univariate and multivariable analyses, Fig 6C and 6D). Similar results of Egger’s test were identified in the majority of the clinicopathological characteristics; a significant publication bias was only identified in lymph node metastasis (P<0.001, data not shown).

**Fig 7:**
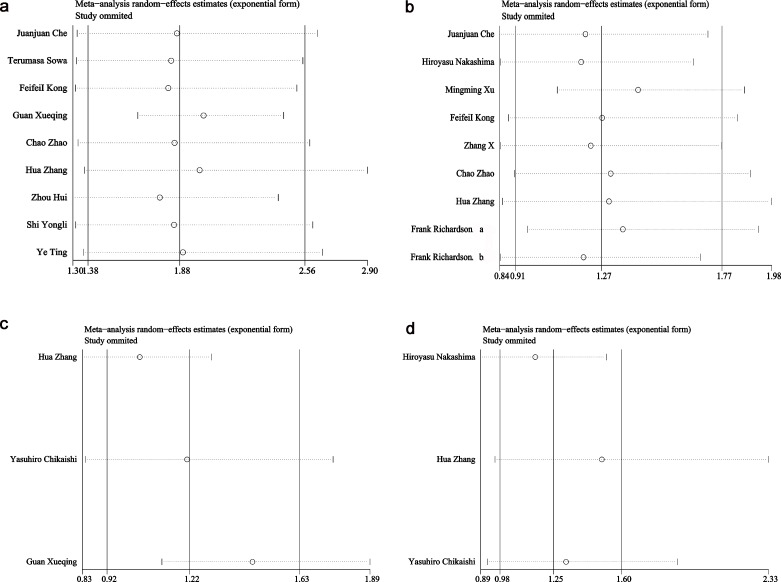
Sensitivity analyses of included studies. (a) OS in univariate analysis, (b) OS in multivariate analysis, (c) DFS in univariate analysis, and (d) DFS in multivariate analysis.

The results of the sensitivity analysis are shown in Fig 7. No significant changes were identified in the pooled results when a study was removed, which indicated that the results of this meta-analysis were reliable.

## Discussion

Metastasis, invasion and drug resistance lead to the failure of cancer treatments in patients with NSCLC. Thus, poor survival has remained an issue despite technological advances in treatments. Several biomarkers have been investigated to predict the progression and prognosis; however, stable and reliable markers have not been identified. Therefore, there is a critical demand to investigate novel biomarkers with high sensitivity and specificity with the goal to improve the survival of NSCLC.

Vimentin is an important component of the intermediate filament family and plays a vital role in the anchorage of organelles and cellular integrity[8]. Furthermore, vimentin,as a mesenchymal phenotype, is a sign of the epithelial-to-mesenchymal transition (EMT), which is essential to carcinogenesis[51]. Recently, increasing evidence has demonstrated that vimentin exerted a substantial influence on the progression and prognosis of cancer. In NSCLC, vimentin was involved in various aspects of cancer, including metastasis, drug resistance, EMT and recurrence. Moreover, the prognostic significance of vimentin has been investigated. However, the results among previous studies have been inconsistent.

The conclusion of several published studies indicated that vimentin was significant in predicting the prognosis and progression of NSCLC. However, inconsistent results have also been demonstrated in published studies. Therefore, a cumulative and combined analysis of these studies was necessary to determine the value of vimentin in the prognosis and progression of NSCLC. The current study comprises the first meta-analysis to assess the value of vimentin in predicting the progression and prognosis of NSCLC. In the univariate analysis of the OS, the pooled HR indicated that positive vimentin expression predicted a shorter OS. Nevertheless, in the multivariate analysis, in which the analysis removes confounding factors, the pooled HR of the OS failed to indicate a significance of vimentin in the prognosis of NSCLC. In this meta-analysis, univariate analysis and multivariate analysis showed inconsistent trend of prognostic values of vimentin expression in NSCLC. The conflict between the two analyses may depend on the less number and samples. Prospective studies with large sample sizes are required to confirm the value of vimentin in the prediction of prognosis and progression in NSCLC. Moreover, the pooled HR of the DFS suggested that vimentin was not an independent prognostic factor in the univariate or multivariate analyses. The current meta-analysis results also indicated that up-regulated vimentin predicted poor differentiation of cancer cells, advanced TNM stages, vascular invasion, lymph node metastasis and recurrence; moreover, in NSCLC with smoking or squamous cell carcinoma, vimentin expression was also up-regulated compared with cases with no smoking or adenocarcinoma. Another interesting finding of this paper that the overexpression of vimentin was no significant depended the age, gender, diameter, T stage, distant metastasis, or marginal invasion! But literature reviews suggest that overexpression of EMT-TFs factors (e.g.vimentin) have a significate correlated to another cancer, like MBC, liver cancer. The cause may be that although EMT-TFs factors (e.g.vimentin) have been demonstrated to be vital in the carcinogenesis of various cancers, the aberrant expression of vimentin and its mechanisms may be different in different cancers because of the heterogeneity of cancers. Thus, the associations between vimentin and clinicopathological characteristics in NSCLC may be inconsistent to other cancers, like MBC, liver cancer. Therefore, vimentin was closely related to the progression of NSCLC and predicted the progression and prognosis of NSCLC. However, prospective studies with large sample sizes are needed to confirm the significance of vimentin in NSCLC.

Regarding the relationships between vimentin and the progression and prognosis of NSCLC, evidence regarding the molecular mechanism supported the results of this meta-analysis. He et al reported that vimentin overexpression and the EMT were induced by metadherin (MTDH) via the Wnt/β-catenin pathway in lung cancer, which is essential to the invasion and metastasis of cancer progression and may support the results of this meta-analysis that vimentin was associated with vascular invasion and lymph node metastasis in NSCLC[52]. In addition, miRNA-214 up-regulated vimentin expression and promoted the EMT, which resulted in the metastasis of lung adenocarcinoma via suppressor-of-fused protein, and the decreased expression of miRNA-30c may induce the EMT and result in the invasion of NSCLC [53, 54]. Moreover, Wang et al demonstrated that 51 altered miRNAs were identified after the EMT was induced by transforming growth factor beta-1 in lung cancer in vitro, which indicated the potentially critical role of miRNAs in the regulation of vimentin and the EMT in lung cancer [55]. Cancer stem cells, which comprise a small proportion of tumor cells, are undifferentiated and responsible for tumor spreading. Recent studies have demonstrated that several markers of cancer stem cells in lung cancer, such as CD44, CD133 and ALDH1, are closely correlated with vimentin expression and the EMT in lung cancer[26, 56]. Zhao et al. demonstrated that increased vimentin expression and the EMT were induced by cigarette smoke extracts, which may provide an explanation for the high expression in NSCLC patients who smoke [57]. Thus, vimentin contributed to the progression of NSCLC through the EMT.

Four studies did not provide sufficient data to calculate the HR and CI of vimentin in the prediction of prognosis in NSCLC [29, 58–60]. These studies all indicated that vimentin has no significant value in the prediction of OS in lung cancer (all P>0.05). Furthermore, in the studies of Hyojin et al, the results also suggested vimentin did not correlate with DFS in lung adenocarcinoma, and Alex et al. reported that vimentin did not predict progression-free survival (PFS)[58]. The negative results, which were not combined, may influence the pooled results, and the limitation of this meta-analysis will be discussed in the limitation section.

In the past decades, evidence-based medicine has become increasingly valued. A meta-analysis, which combines all the existing evidence and accumulates the individual sample data, can obtain a more stable result and drive a more convincing conclusion, as compared to individual study with small sample size. In this present meta-analysis, we demonstrated that the overexpression of vimentin predicted a poor overall survival of NSCLC in the univariate analysis and up-regulated vimentin expression was associated with several clinicopathological factors, including histology of squamous cell carcinoma, smoking, poor differentiation, advanced TNM stages, vascular invasion, lymph node metastasis and recurrence in NSCLC. By accumulating the individual studies, the results of this meta-analysis were more convincing and could provide more powerful evidence for NSCLC treatment in clinical practice. The current study is the first investigation to focus on the correlations between vimentin and the prognosis and clinicopathological characteristics of lung cancer using a meta-analysis, and the results of this analysis are meaningful. Nevertheless, there were several limitations that must be considered. 1) One of the major limitations of this meta-analysis was that the publication bias tests were underpowered since the studies<10, and it may cause the combined results unstably and affect the conclusion[61]. 2) The studies that did not provide direct data or data that could be used to calculate the HR or were excluded, which may result in an inaccurate pooled HR and OR. 3) Moreover, only studies published in English or Chinese were included, which may result in a potential publication bias. 4) The studies did not assess completely equal cancer types and clinical stages of NSCLC. 5) Finally, there were no identical standards to define positive vimentin expression, which may contribute to the heterogeneity among the studies and thus attenuate the reliability of the pooled results.

## Conclusions

The present meta-analysis demonstrates that the overexpression of vimentin predicts a poor overall survival of NSCLC in the univariate analysis but not the multivariate analysis. Vimentin has minimal value in predicting disease-free survival in NSCLC. However, up-regulated vimentin expression was associated with several clinicopathological factors, including histology of squamous cell carcinoma, smoking, poor differentiation, advanced TNM stages, vascular invasion, lymph node metastasis and recurrence in NSCLC. Overall, Vimentin is essential to progression in NSCLC, and vimentin expression in the tissues of patients with NSCLC predicts progression and prognosis. Nevertheless, additional, prospective studies with large sample sizes are required to confirm the value of vimentin in the prediction of prognosis and progression in NSCLC.

## Supporting Information

S1 File The process of search, data extraction and combination of the meta-analysis. (PPTX)

S2 File The excluded studies of the meta-analysis for reasons. (DOCX)

S3 File The PRISMA flow diagram of the meta-analysis. (DOC)

S1 Table The PRISMA checklist of the meta-analysis. (DOC)
