# Supplementary material for: Prognostic Values of Vimentin Expression and Its Clinicopathological Significance in Non-Small Cell Lung Cancer: A Meta-Analysis of Observational Studies with 4118 Cases
Source: PLoS One. 2016 Sep 22;11(9):e0163162. doi: 10.1371/journal.pone.0163162 (PMC5033348; doi:10.1371/journal.pone.0163162)
Supplement: S1 File — (PPTX) [file pone.0163162.s001.pptx]

## Slide 1
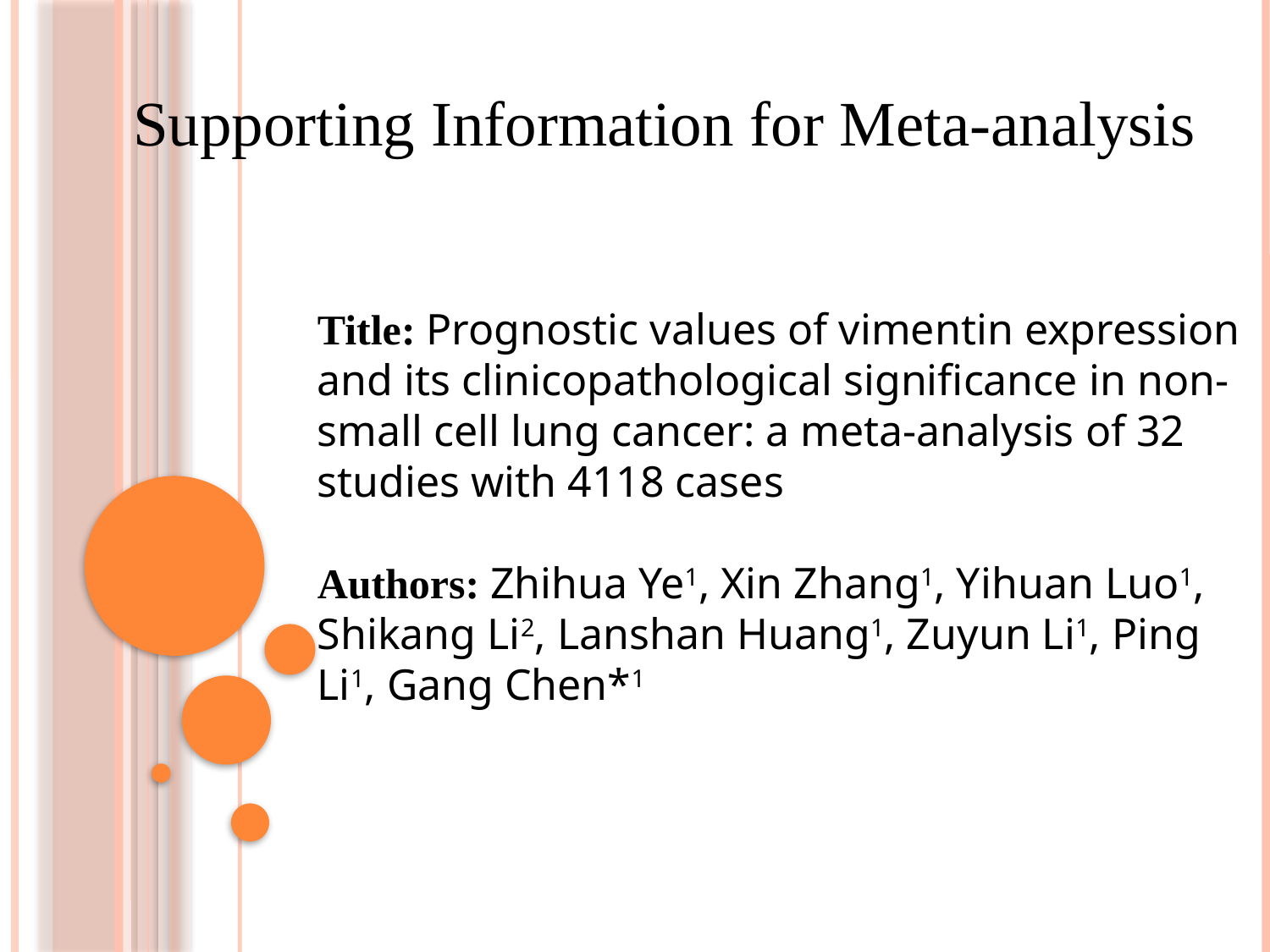

Supporting Information for Meta-analysis
Title: Prognostic values of vimentin expression and its clinicopathological significance in non-small cell lung cancer: a meta-analysis of 32 studies with 4118 cases
Authors: Zhihua Ye1, Xin Zhang1, Yihuan Luo1, Shikang Li2, Lanshan Huang1, Zuyun Li1, Ping Li1, Gang Chen*1

## Slide 2
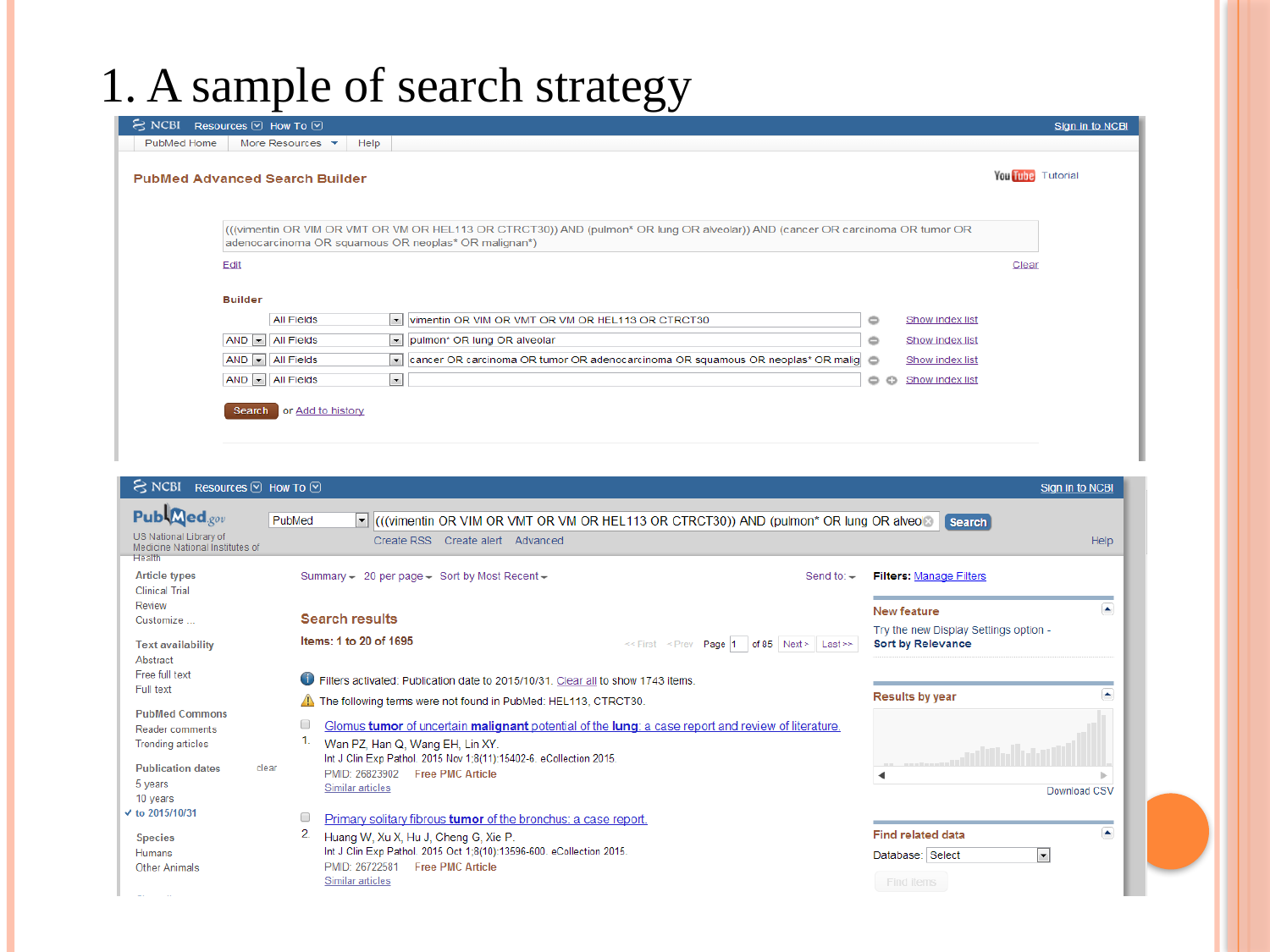

1. A sample of search strategy

## Slide 3
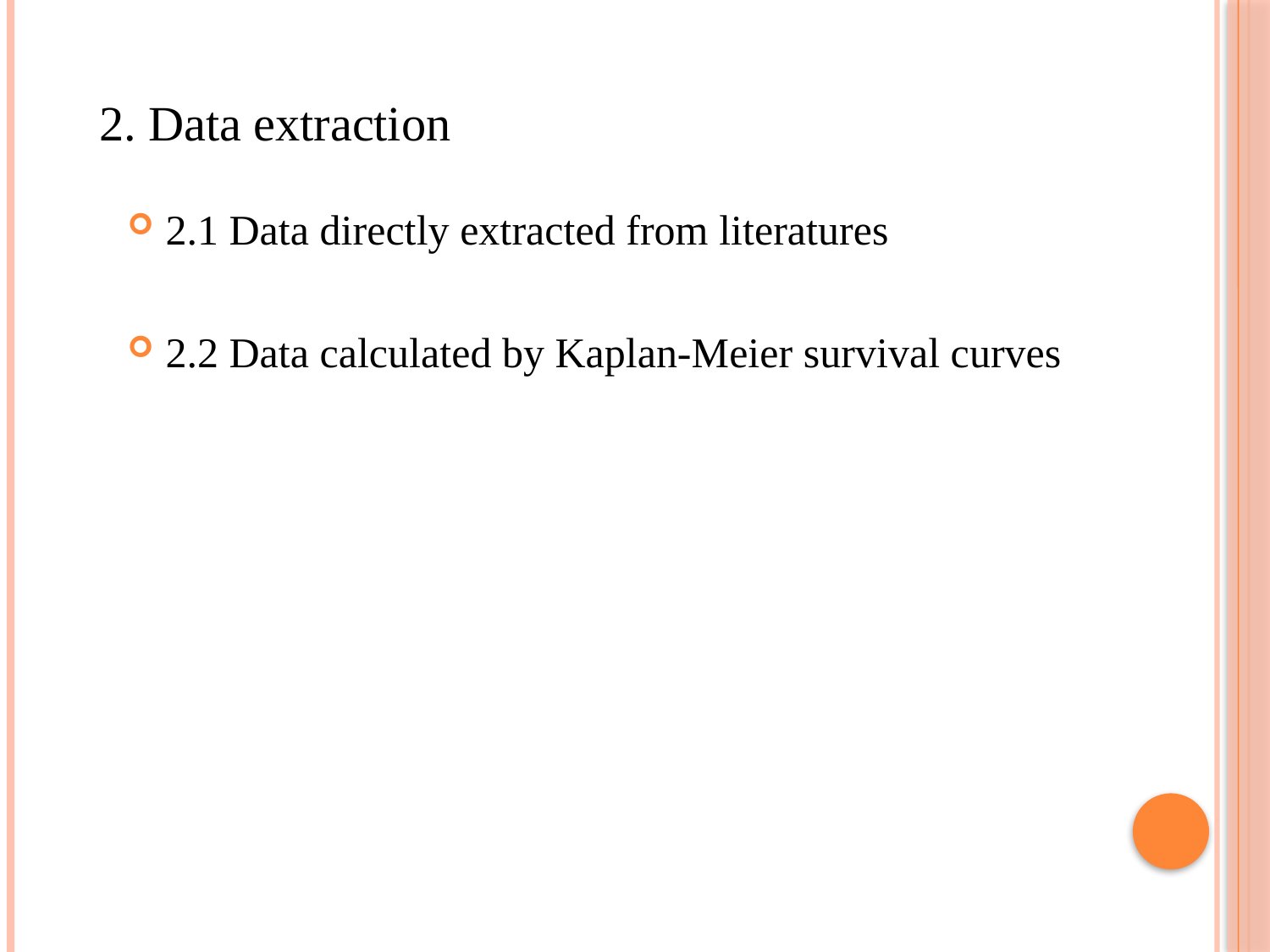

2. Data extraction
2.1 Data directly extracted from literatures
2.2 Data calculated by Kaplan-Meier survival curves

## Slide 4
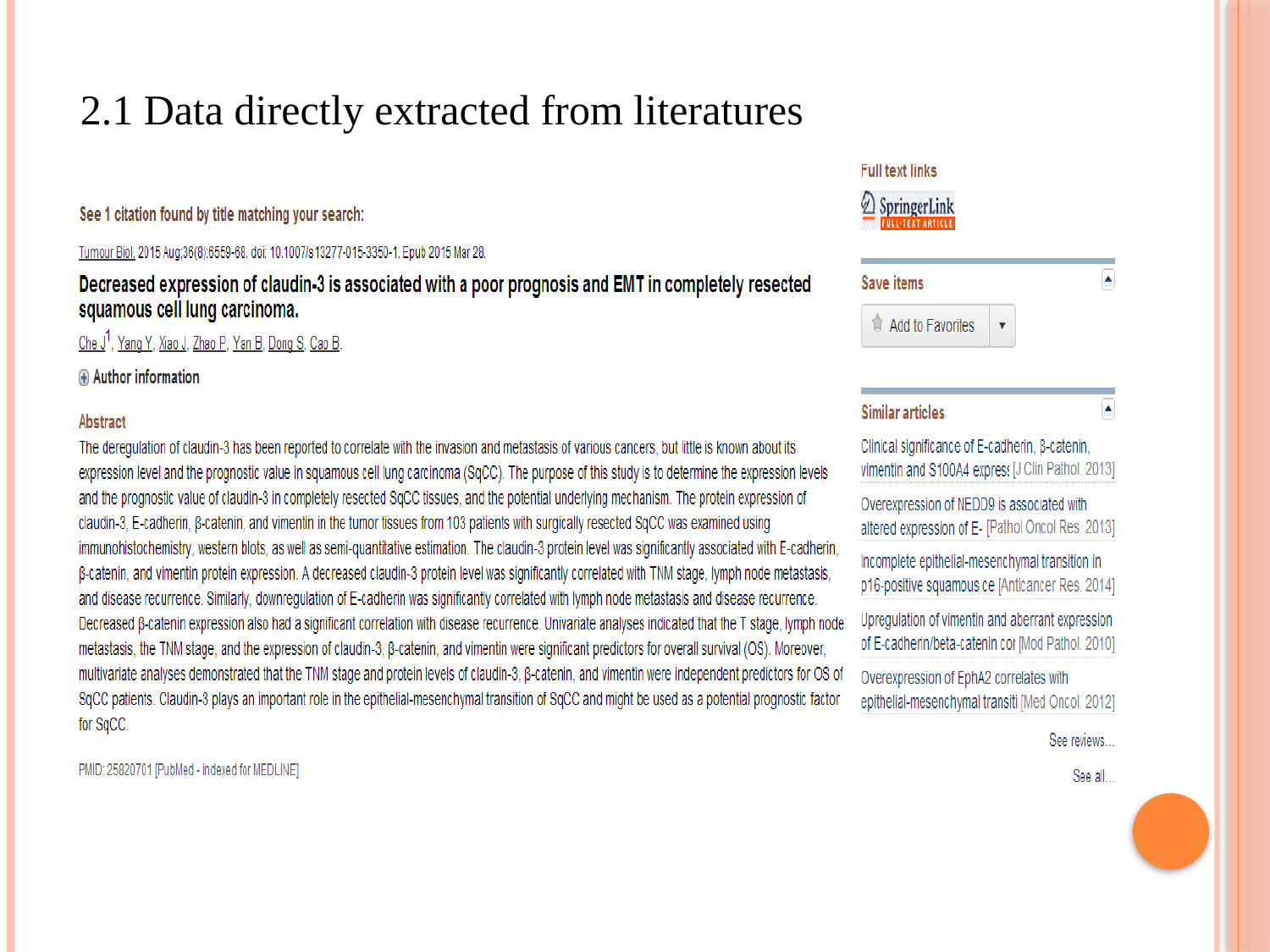

2.1 Data directly extracted from literatures

## Slide 5
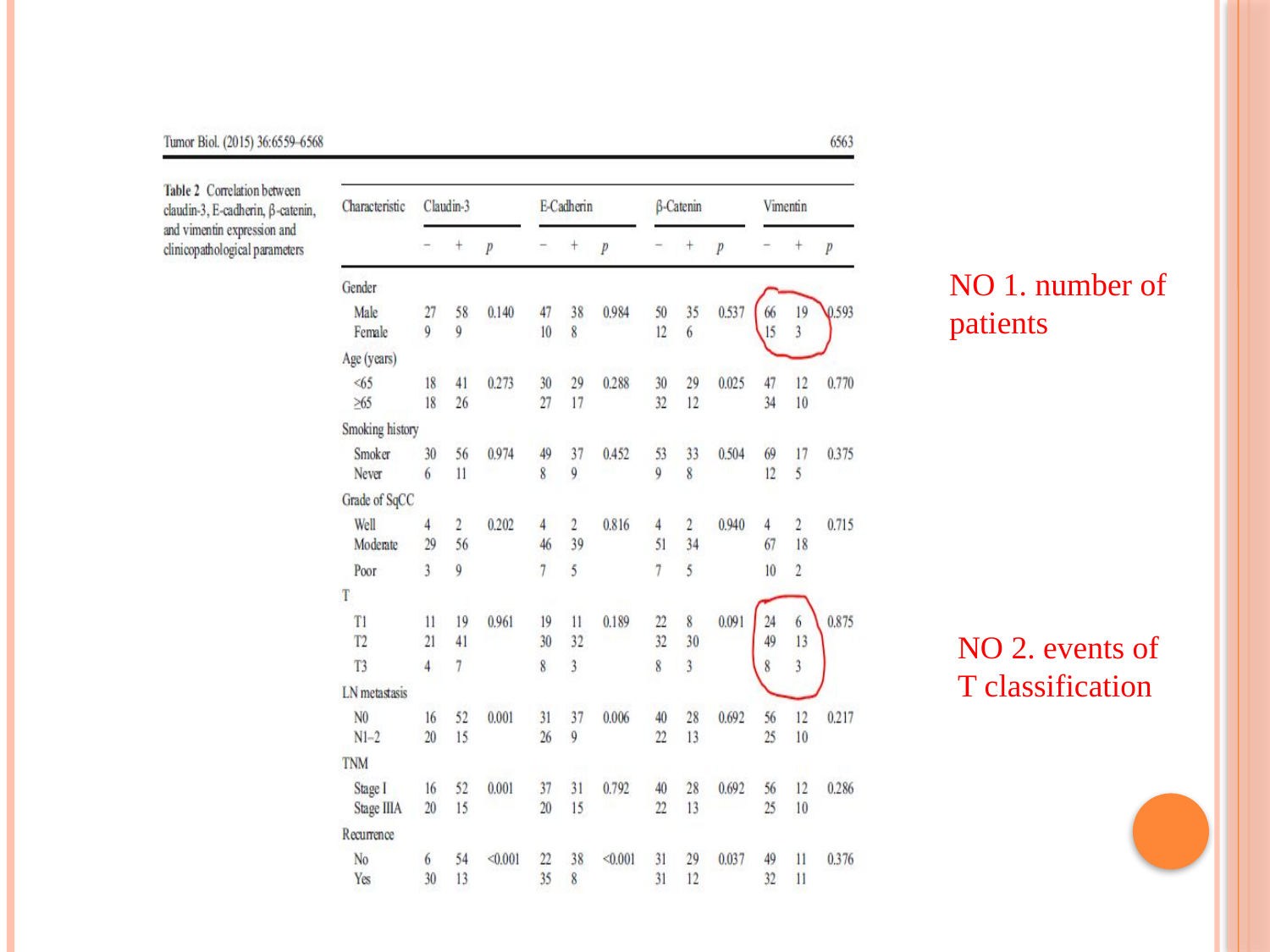

NO 1. number of
patients
NO 2. events of
T classification

## Slide 6
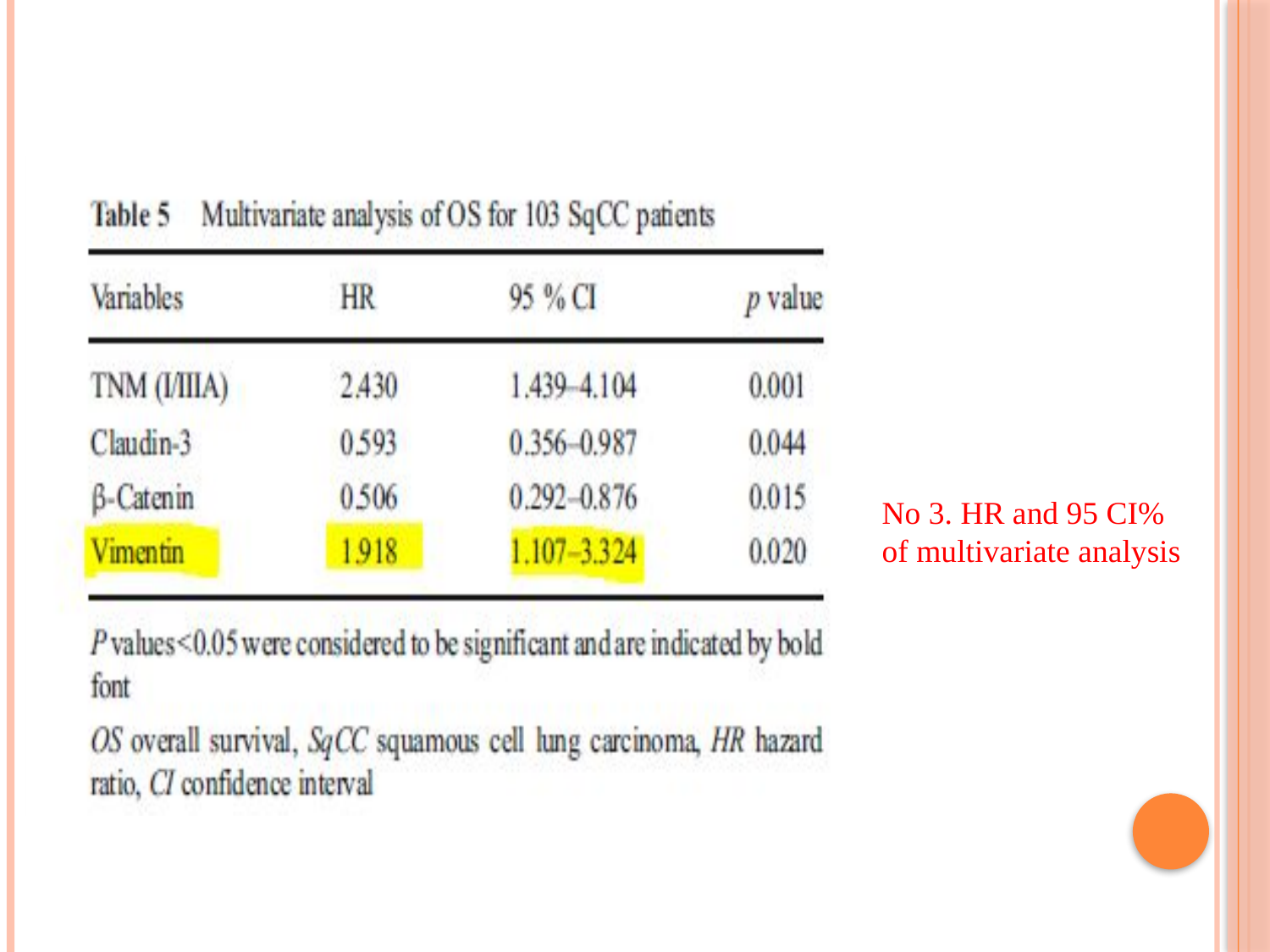

No 3. HR and 95 CI%
of multivariate analysis

## Slide 7
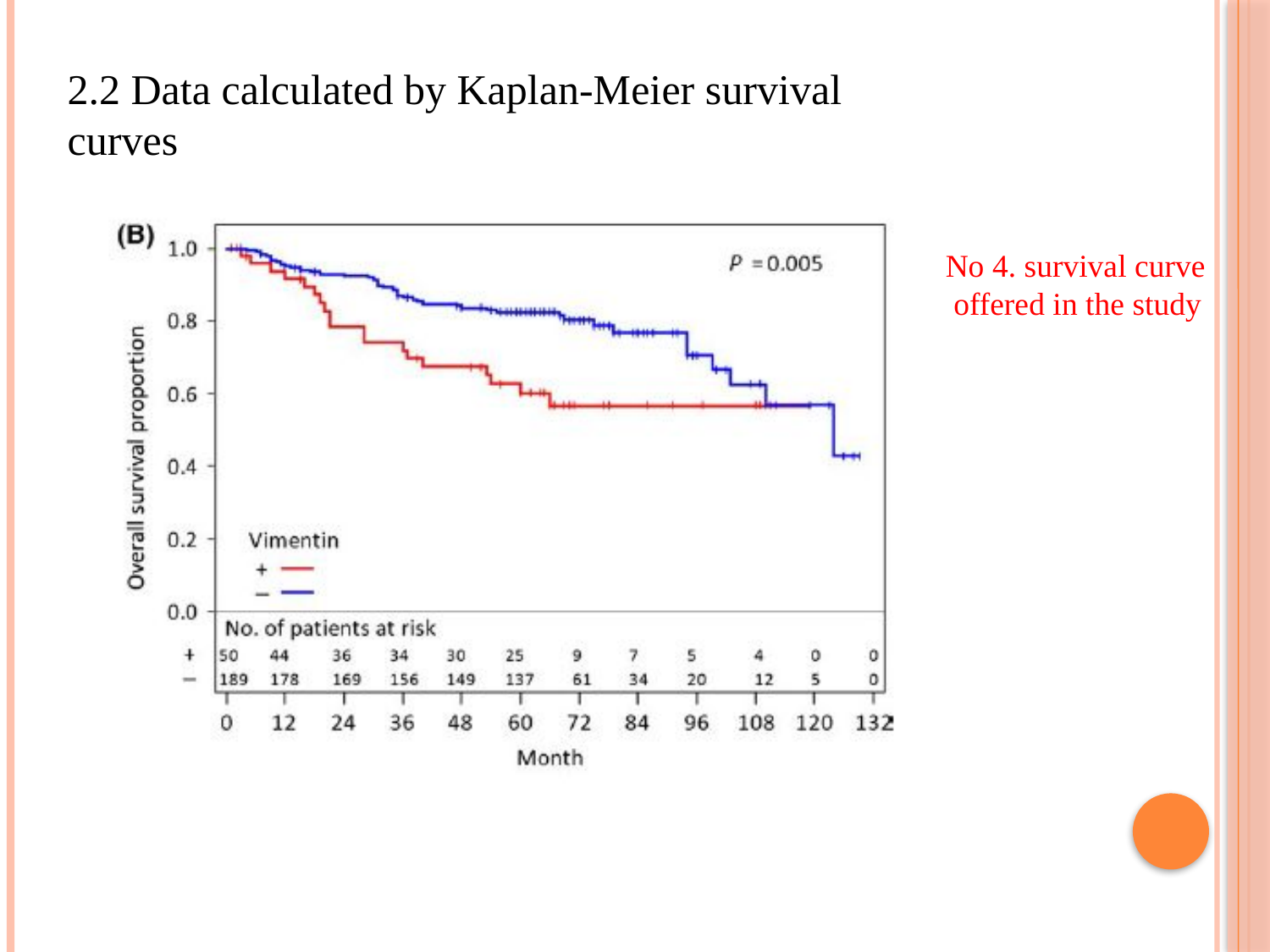

2.2 Data calculated by Kaplan-Meier survival curves
No 4. survival curve
 offered in the study

## Slide 8
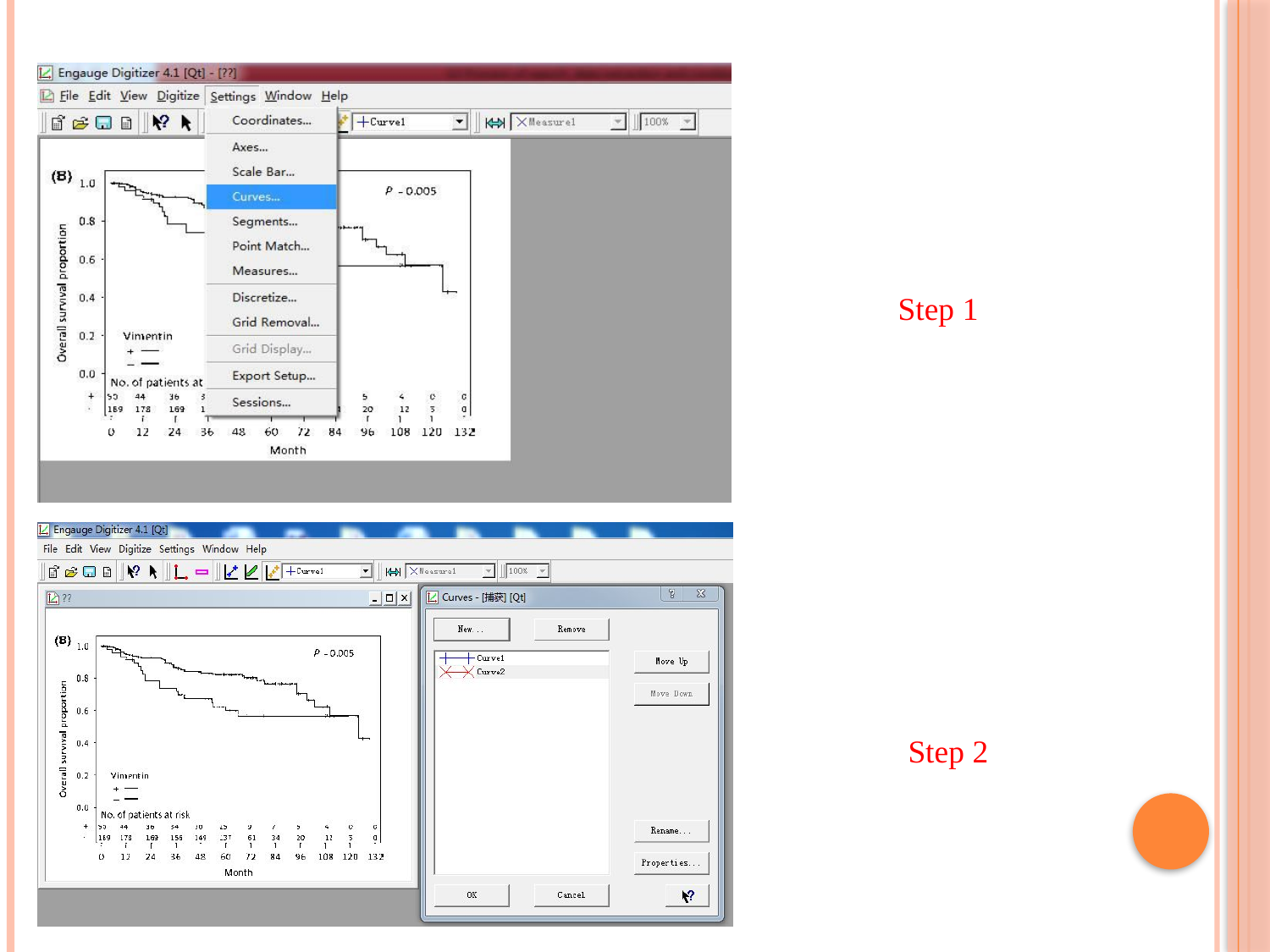

Step 1
Step 2

## Slide 9
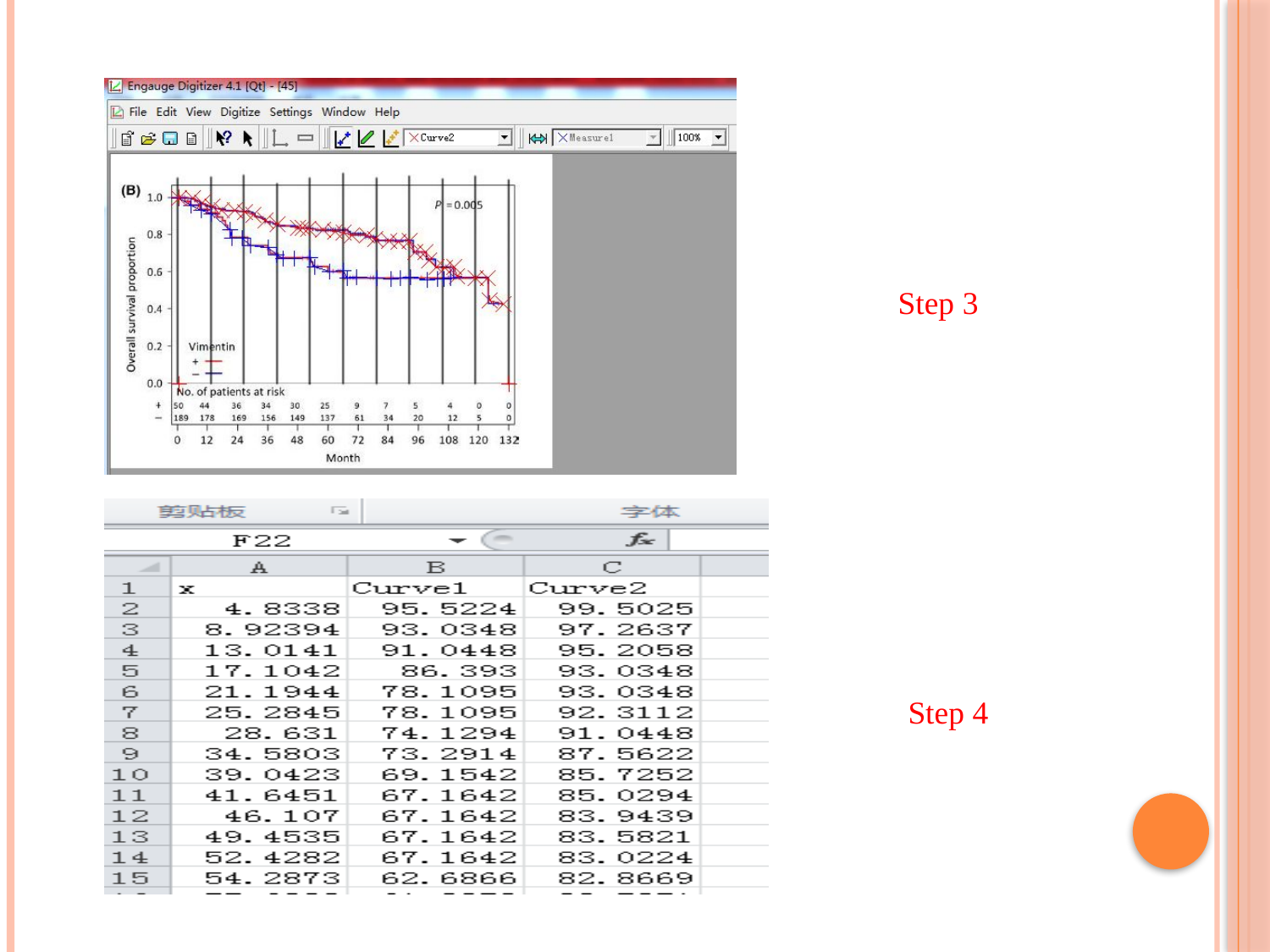

Step 3
Step 4

## Slide 10
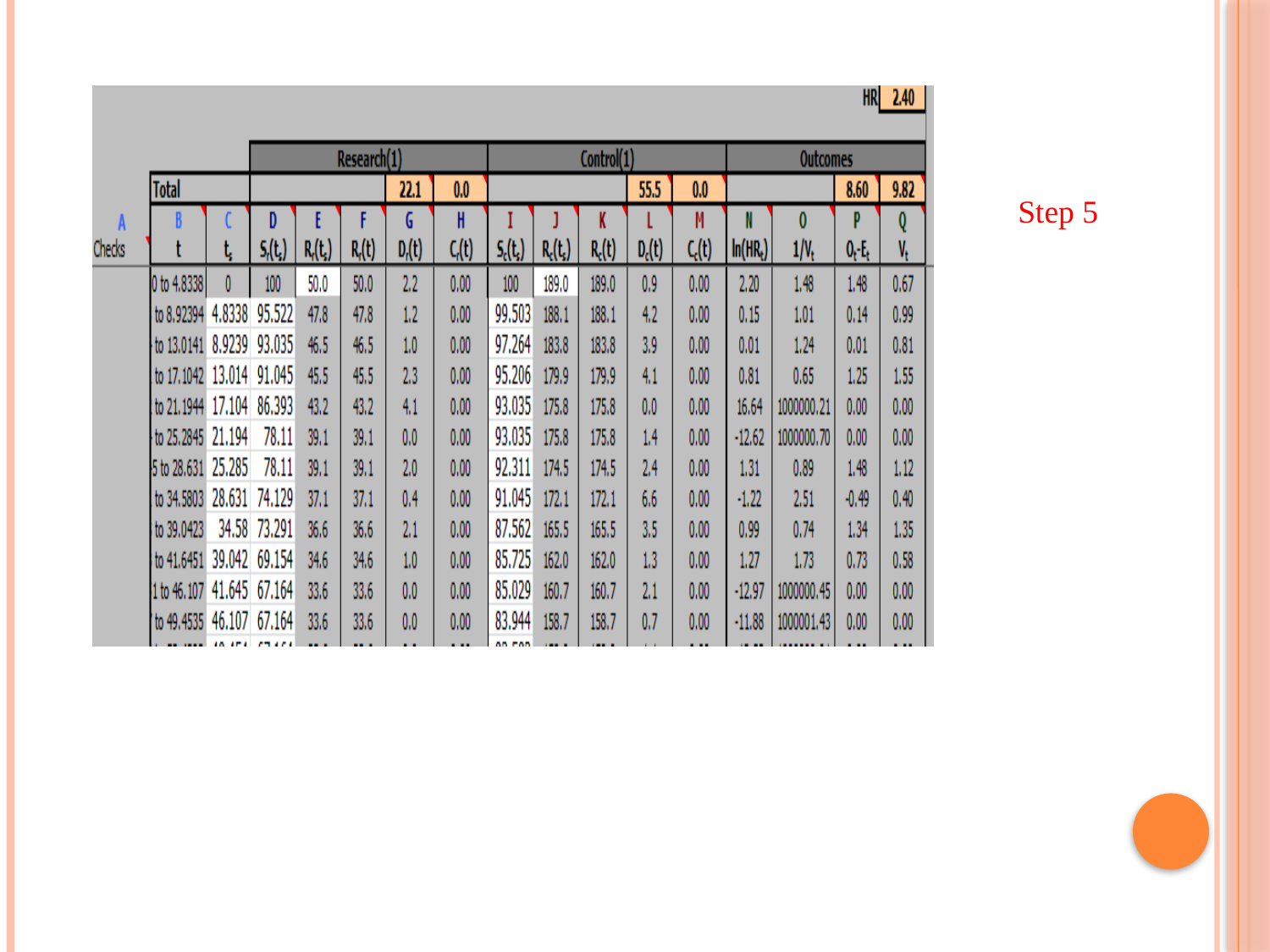

Step 5

## Slide 11
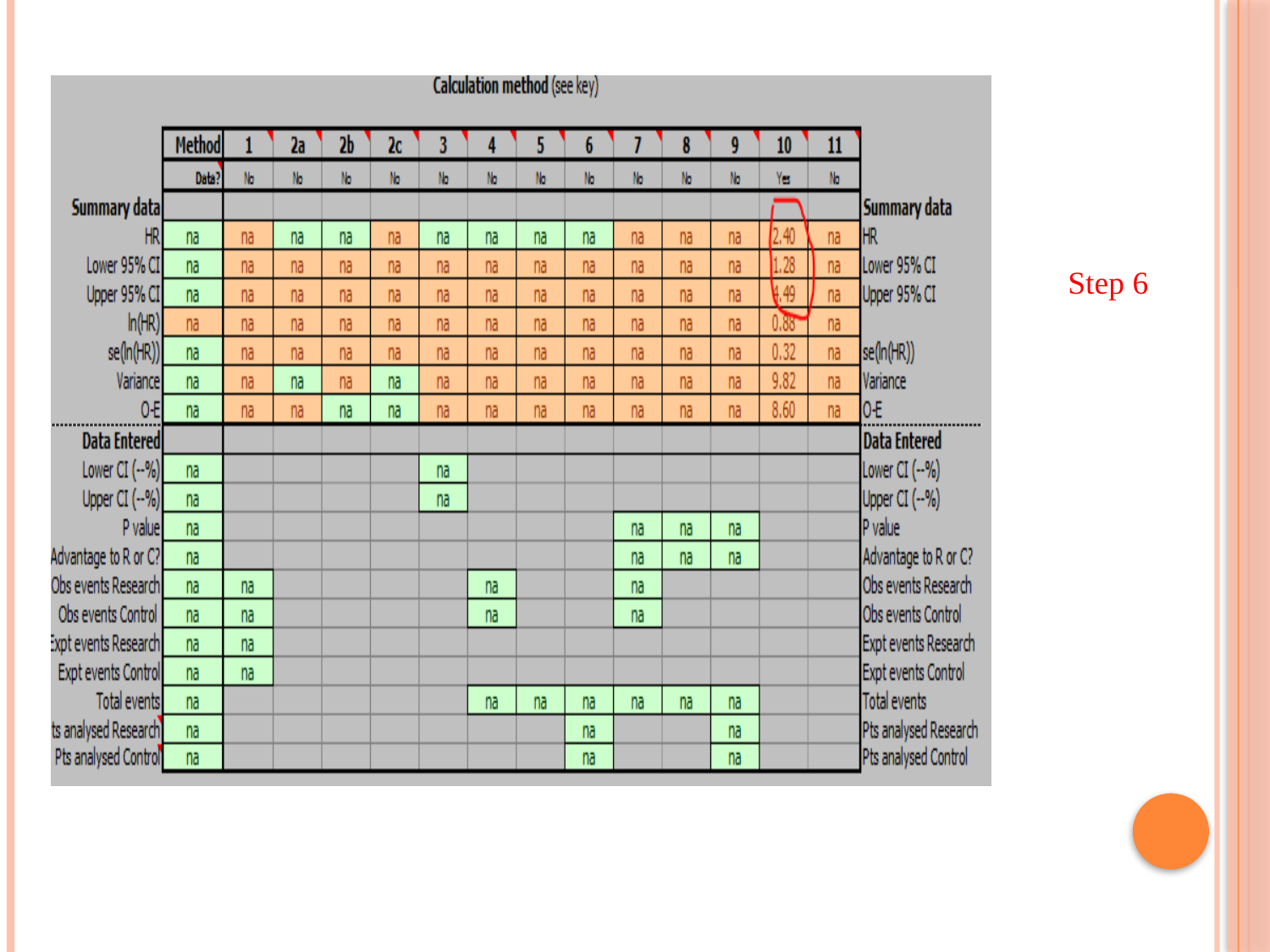

Step 6

## Slide 12
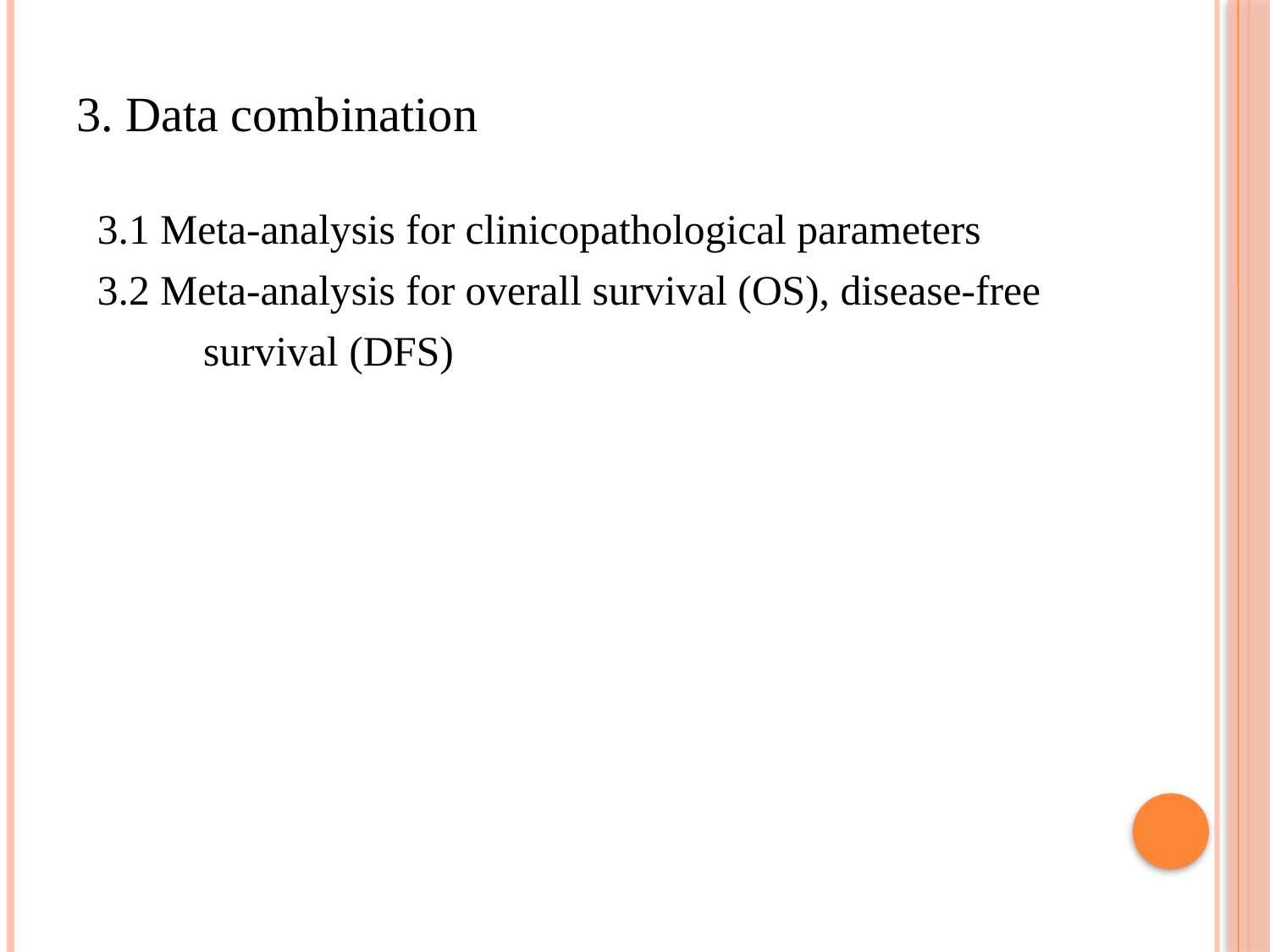

3. Data combination
3.1 Meta-analysis for clinicopathological parameters
3.2 Meta-analysis for overall survival (OS), disease-free
 survival (DFS)

## Slide 13
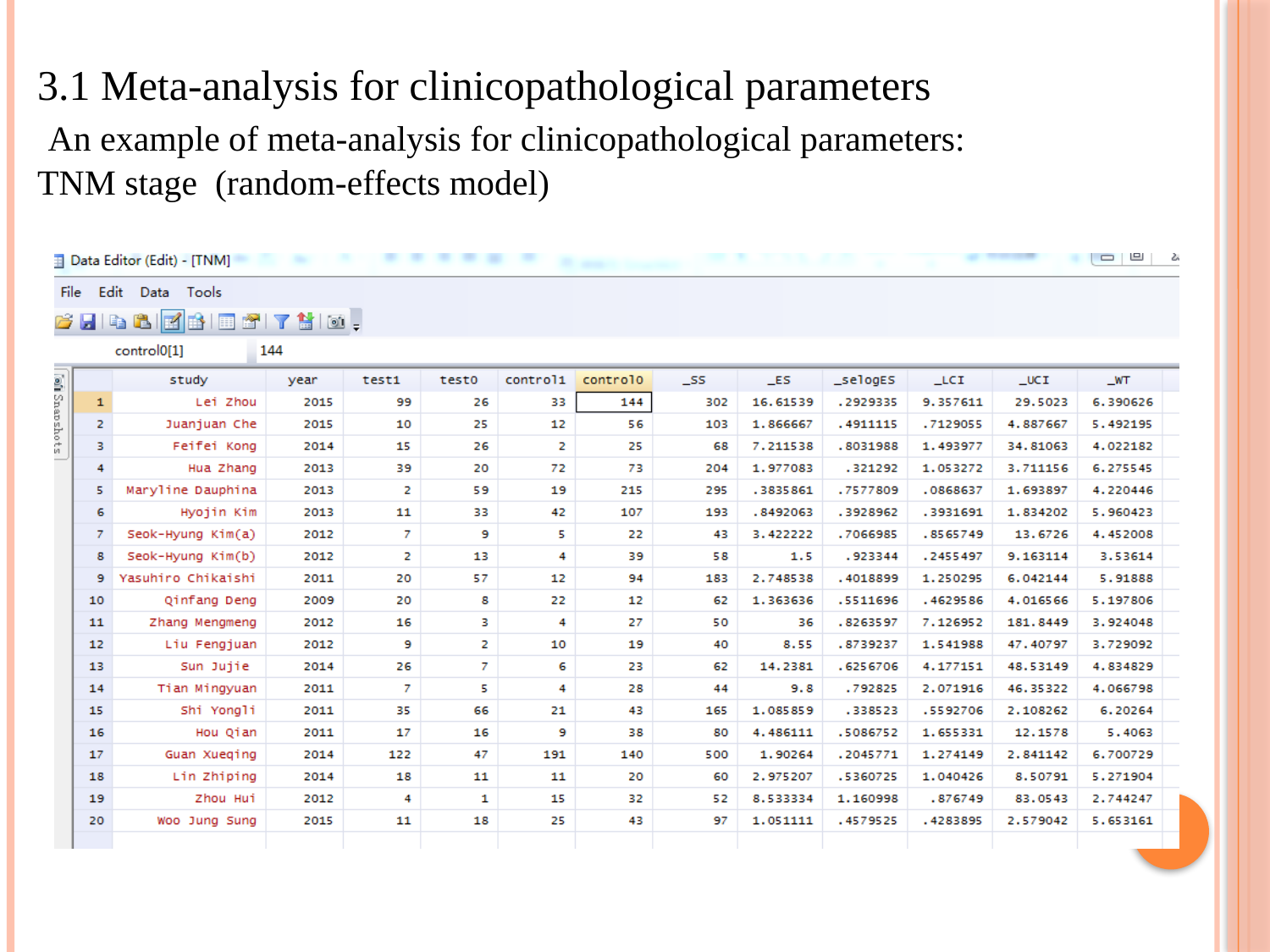

3.1 Meta-analysis for clinicopathological parameters
 An example of meta-analysis for clinicopathological parameters:
TNM stage (random-effects model)

## Slide 14
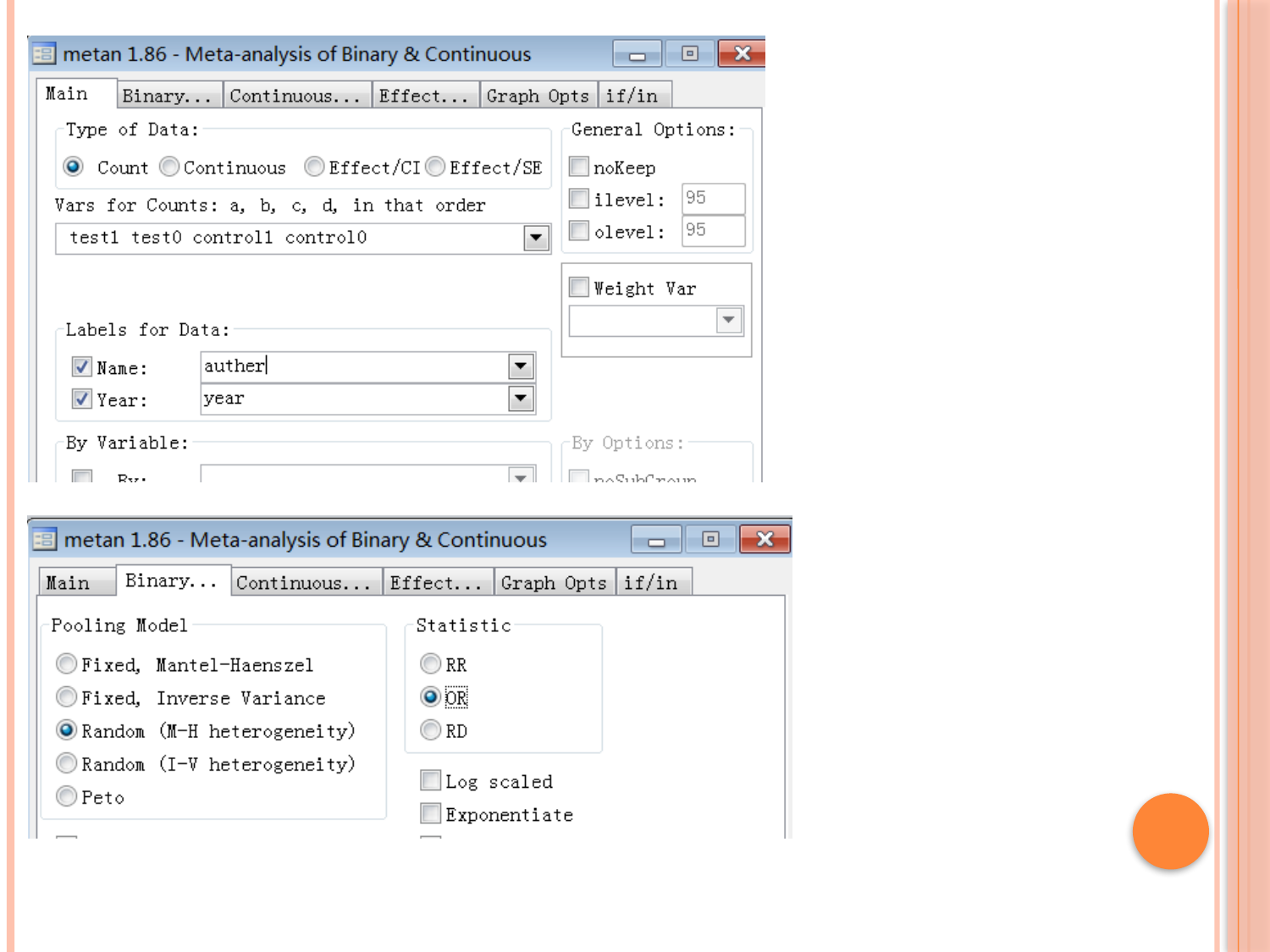

## Slide 15
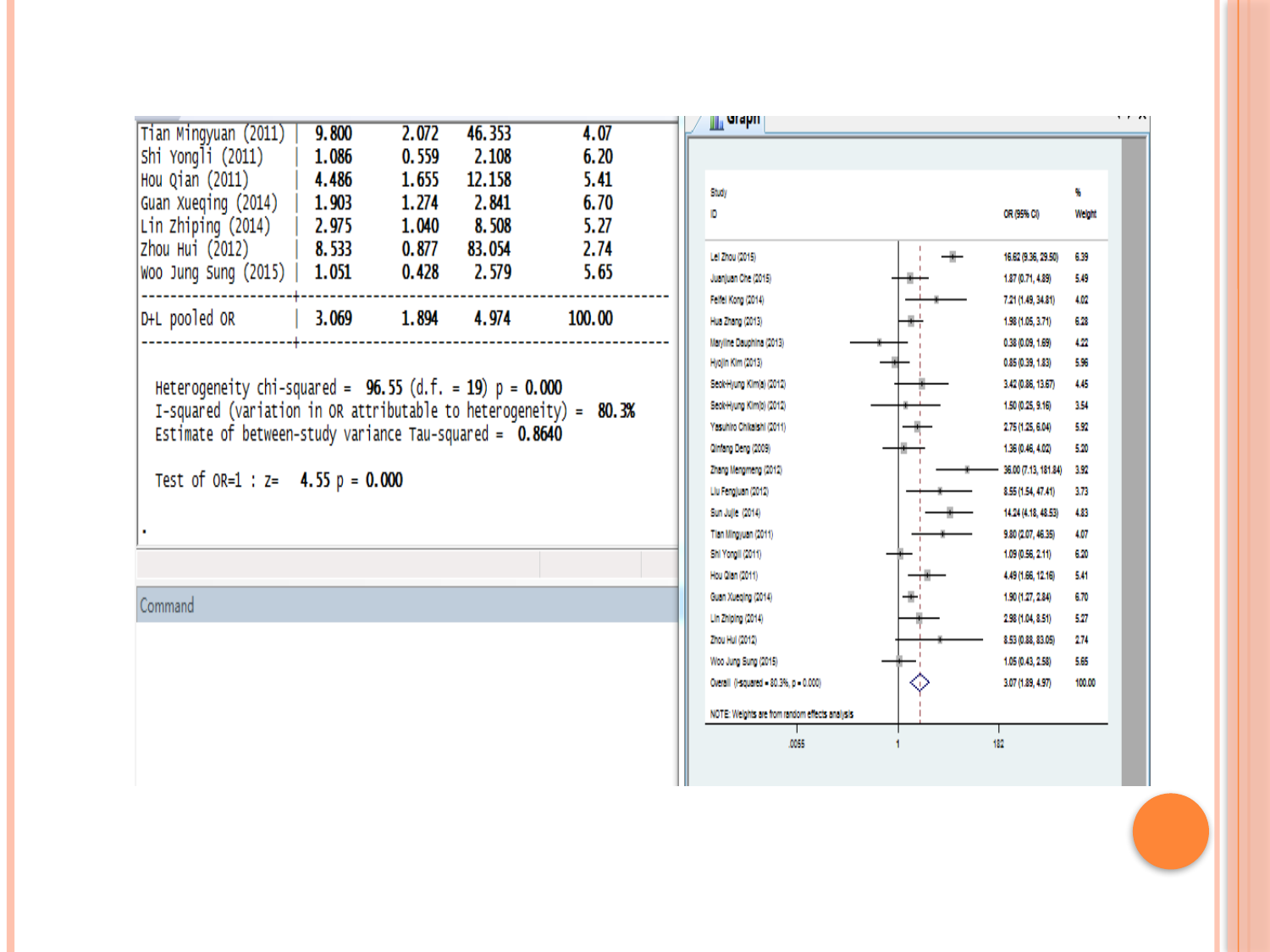

## Slide 16
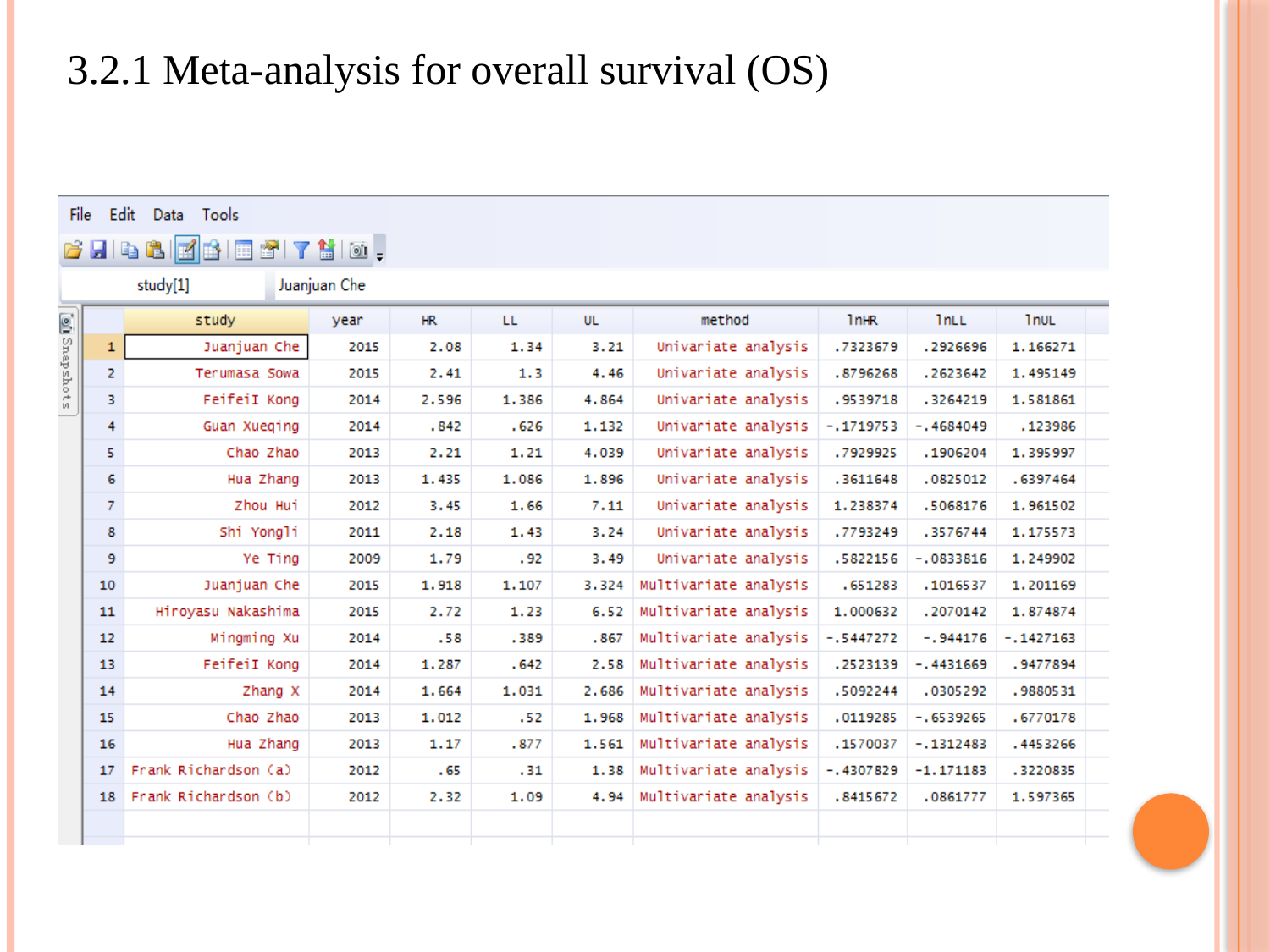

3.2.1 Meta-analysis for overall survival (OS)

## Slide 17
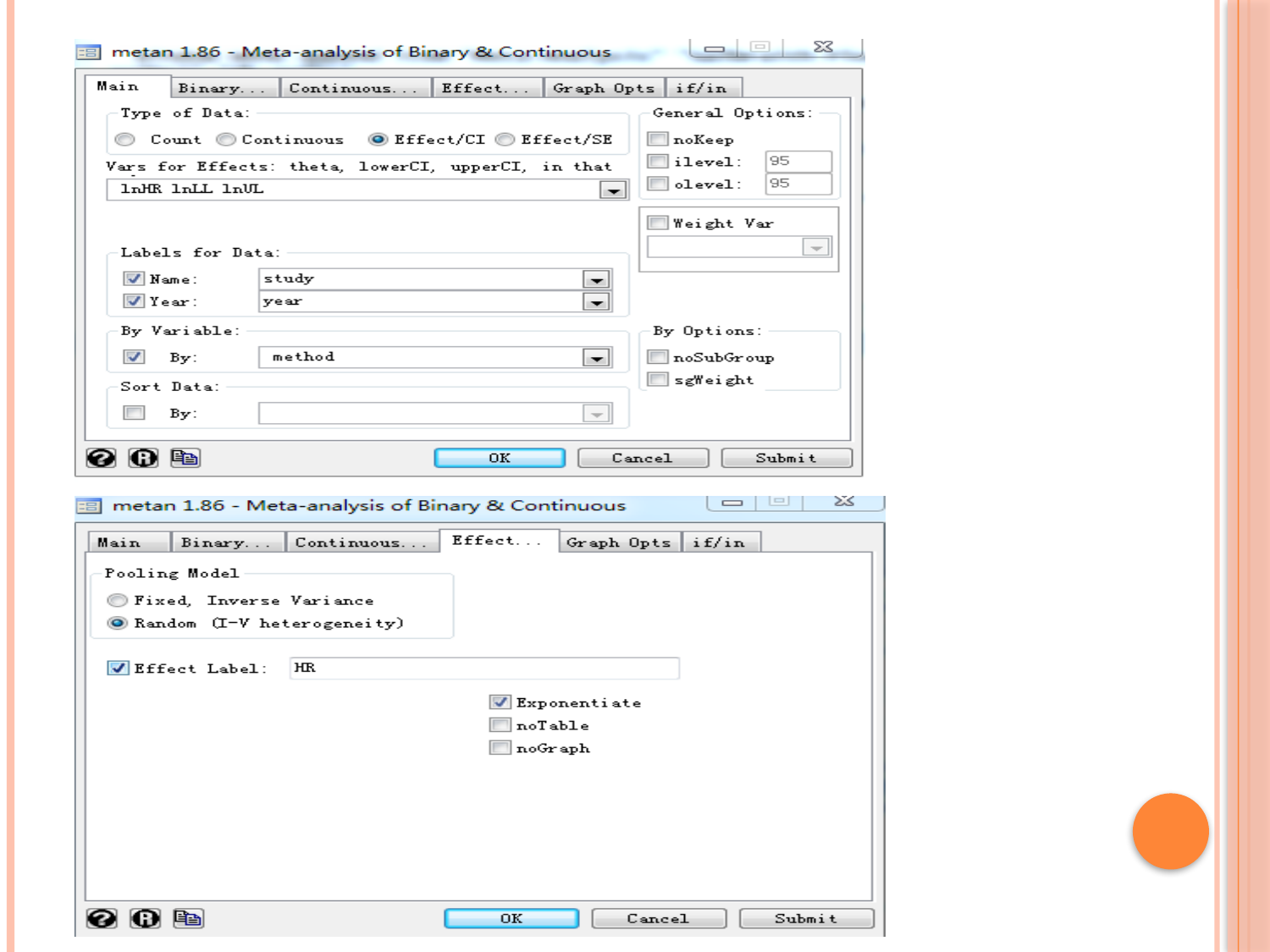

## Slide 18
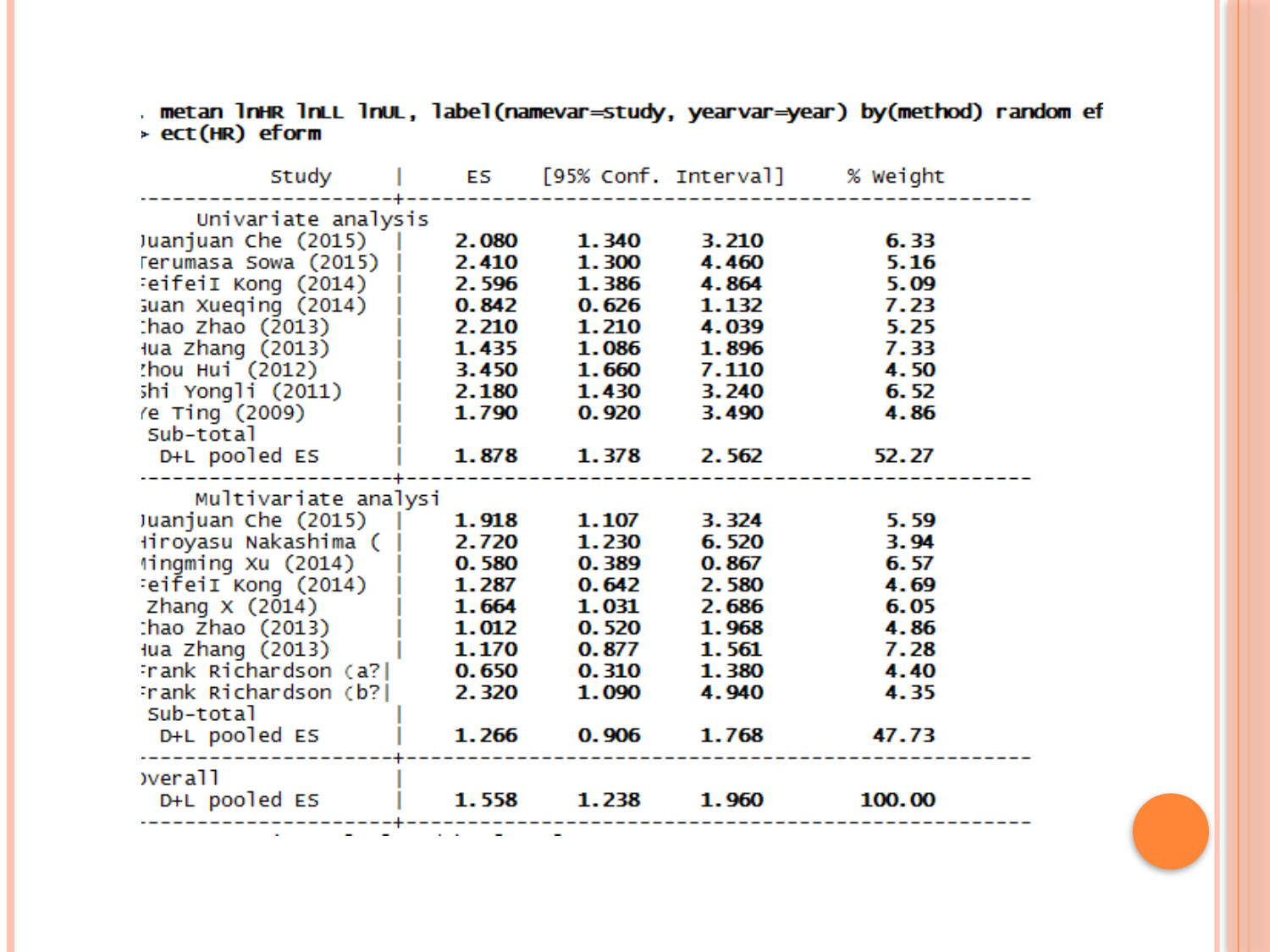

## Slide 19
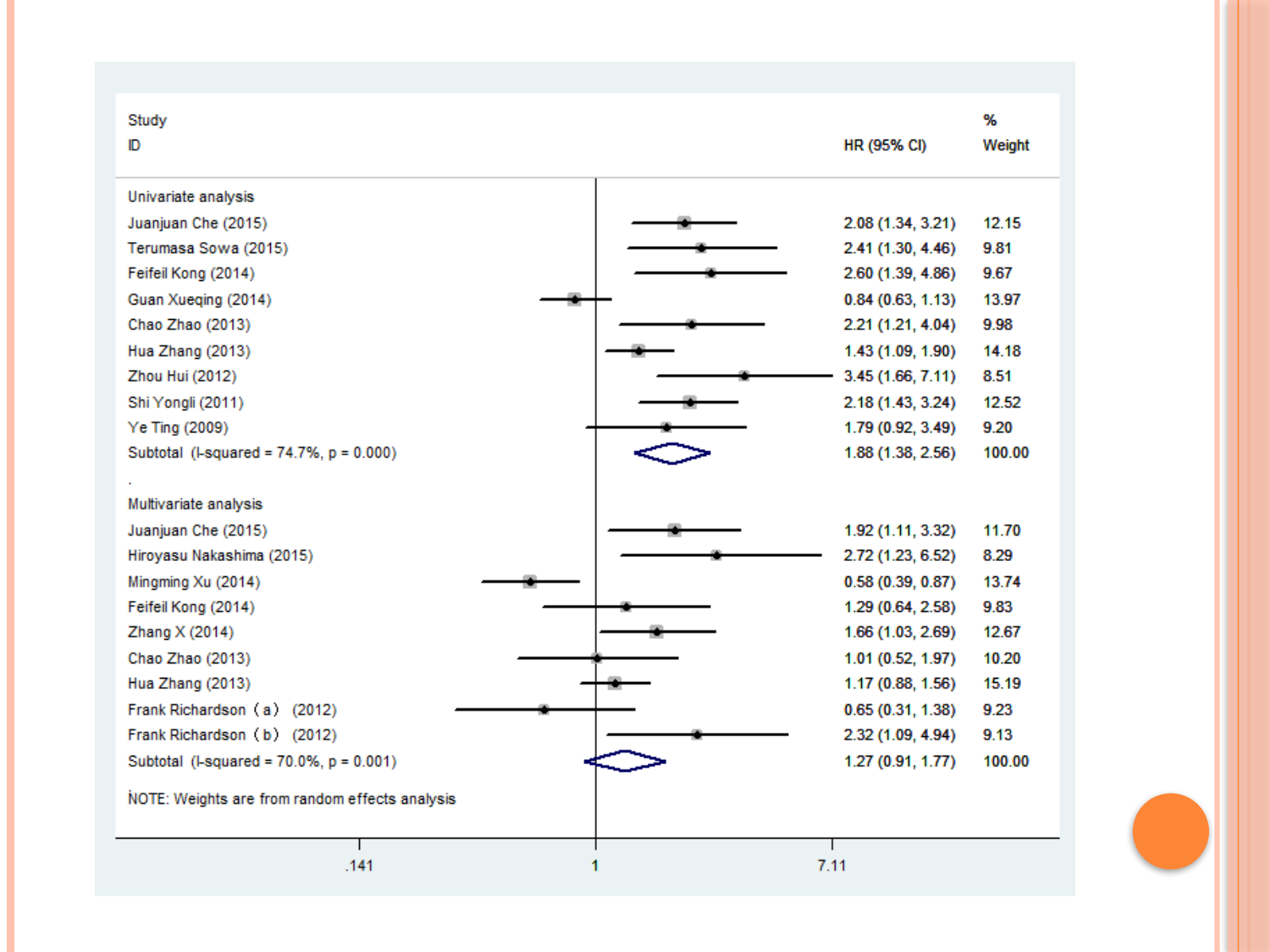

## Slide 20
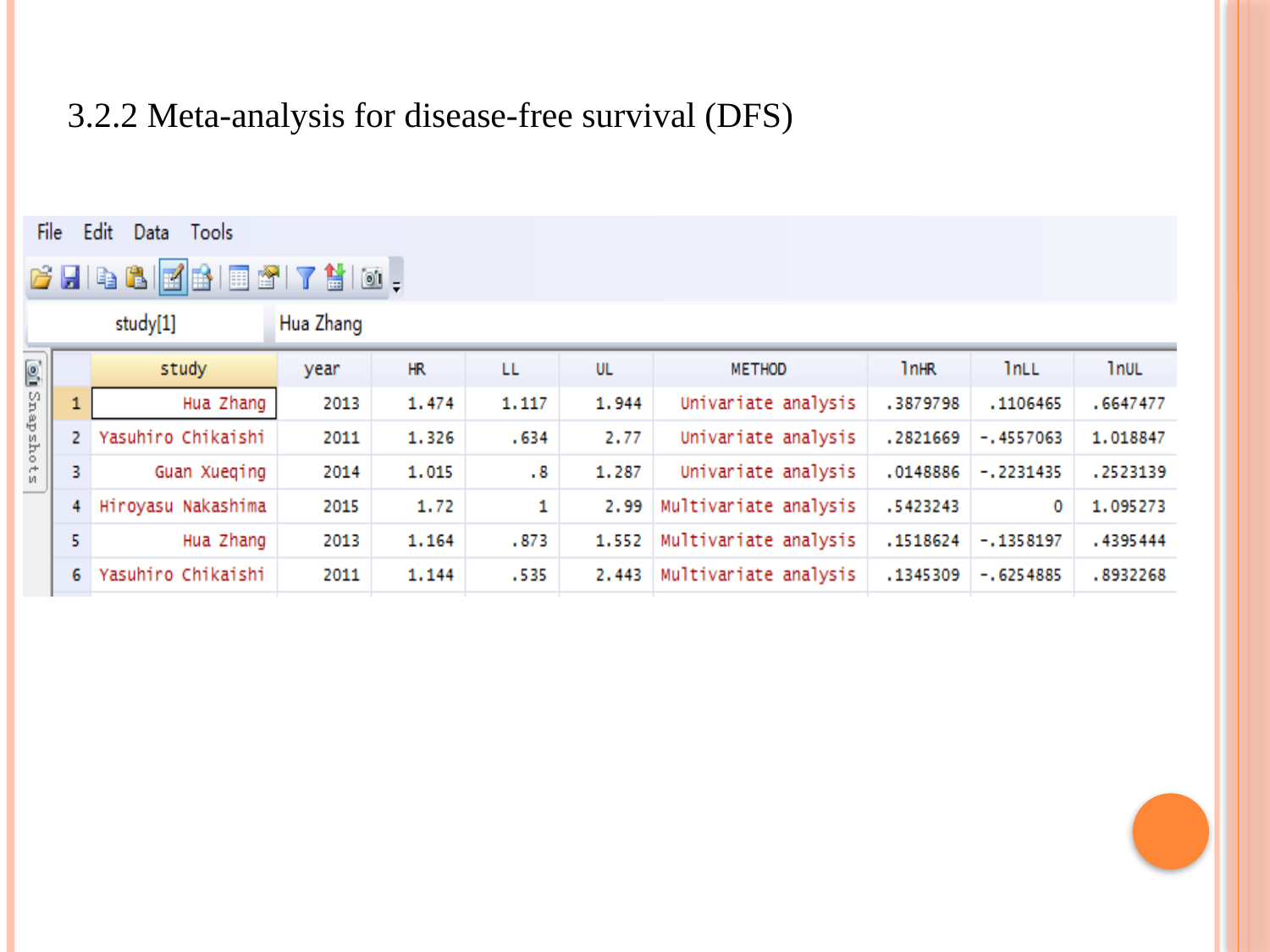

3.2.2 Meta-analysis for disease-free survival (DFS)

## Slide 21
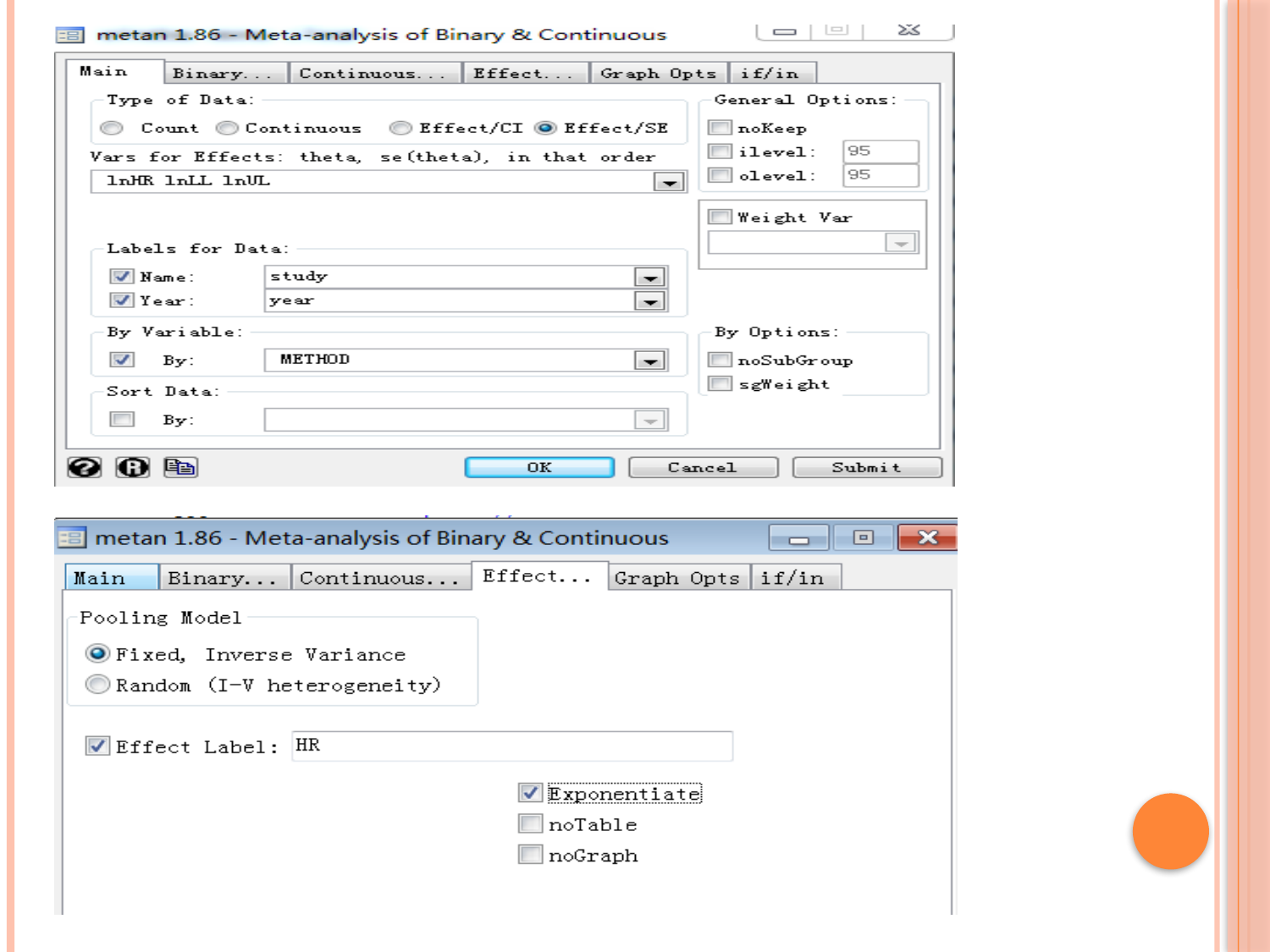

## Slide 22
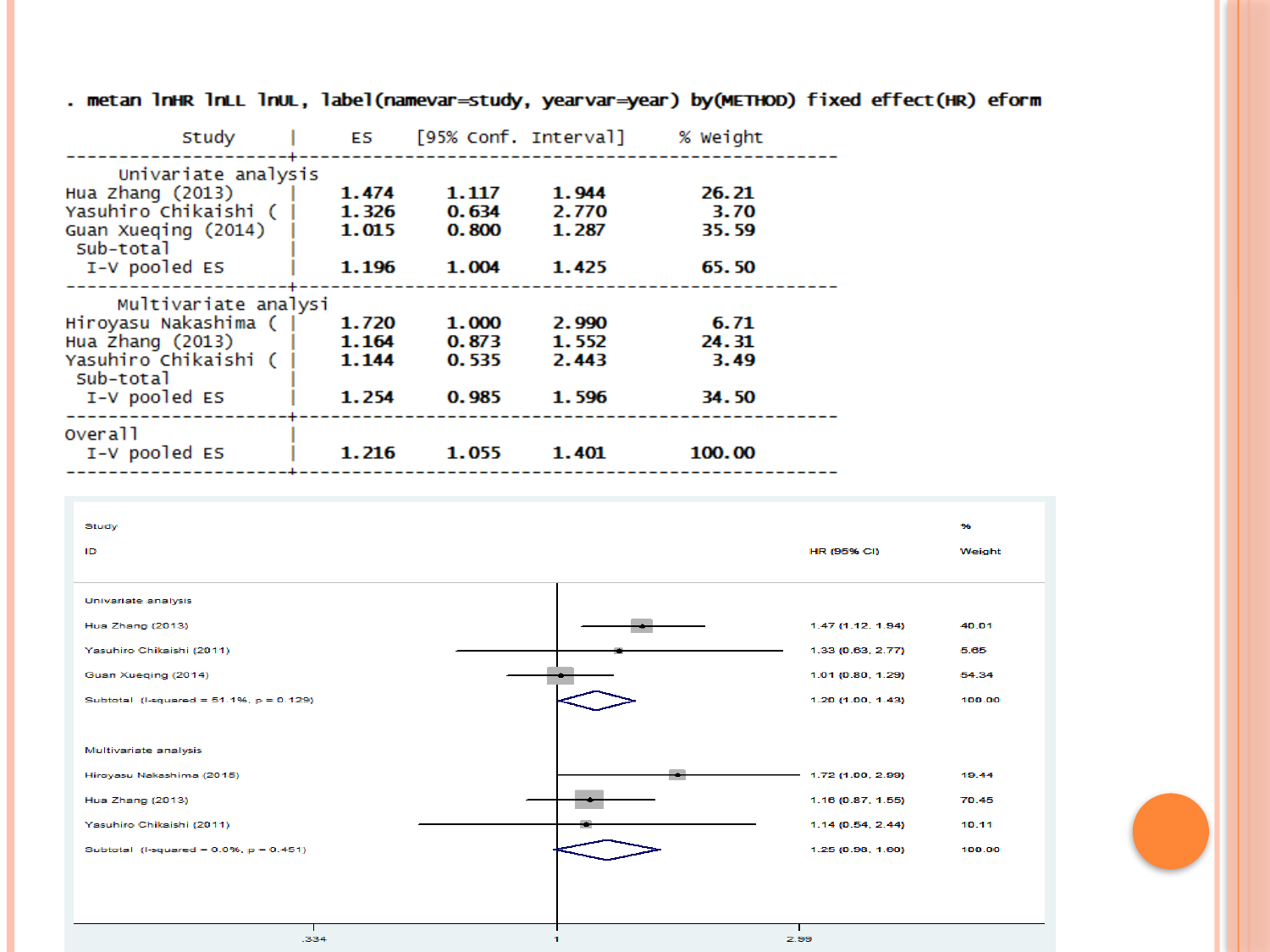

## Slide 23
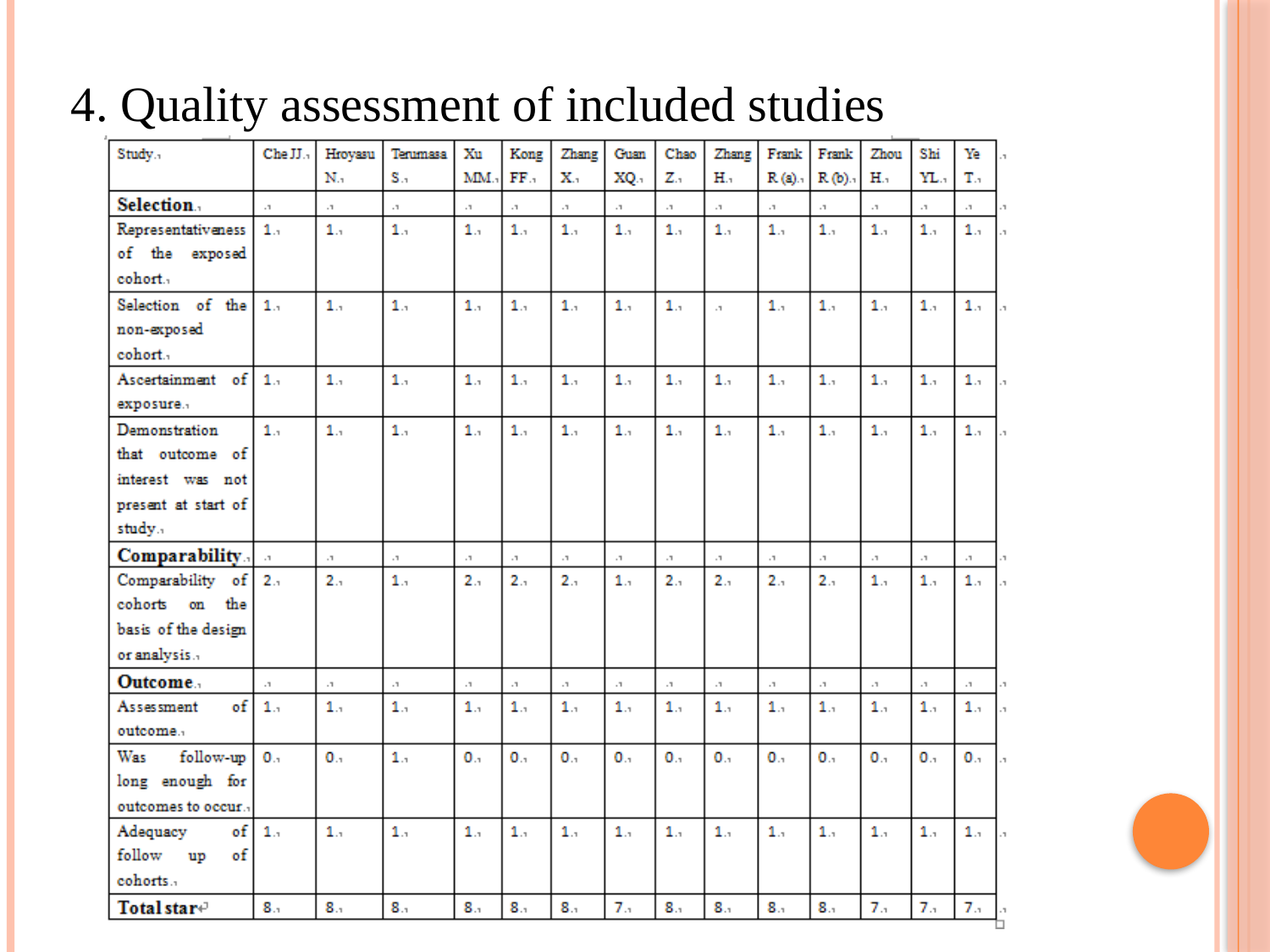

4. Quality assessment of included studies

## Slide 24
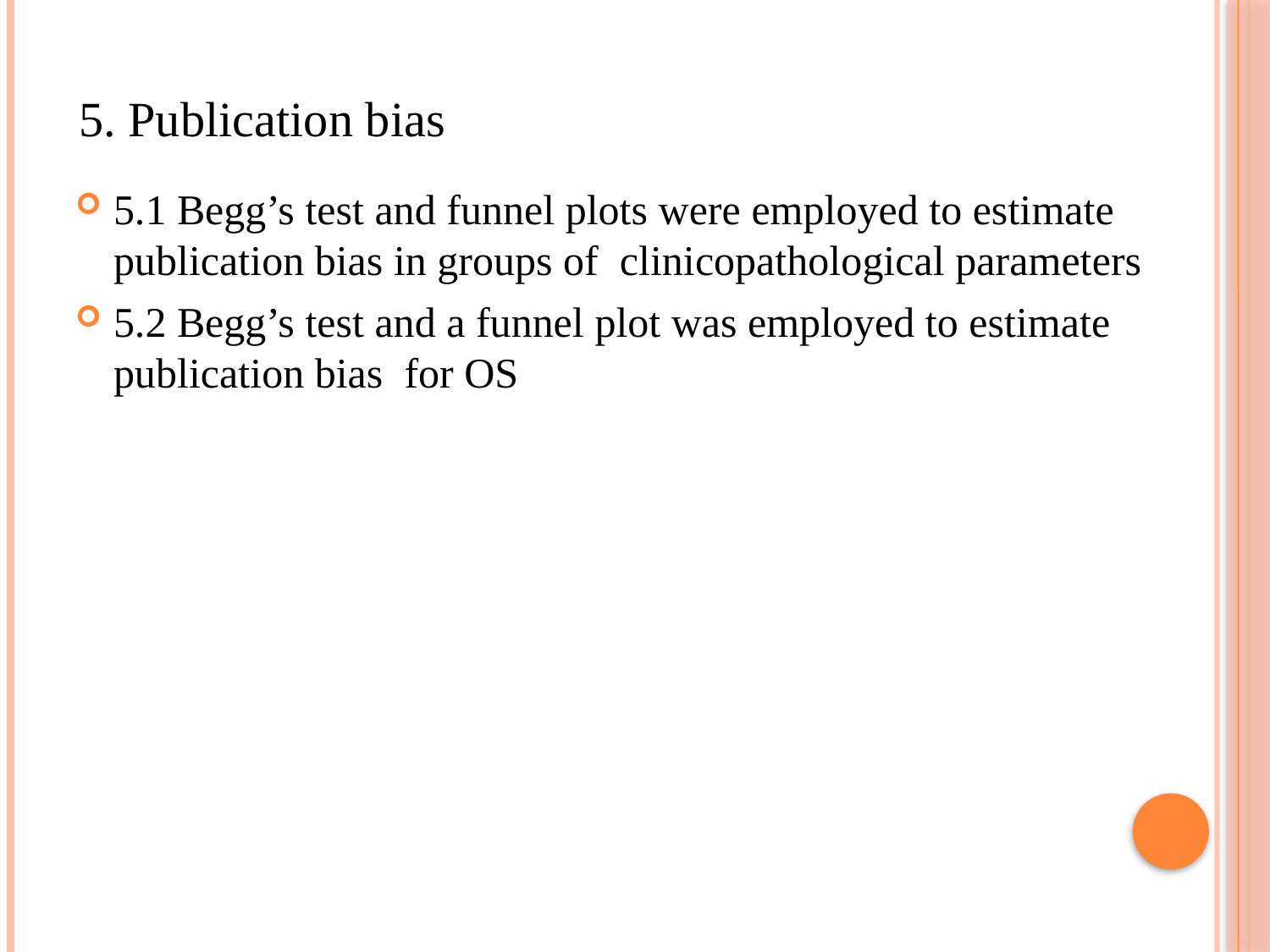

5. Publication bias
5.1 Begg’s test and funnel plots were employed to estimate publication bias in groups of clinicopathological parameters
5.2 Begg’s test and a funnel plot was employed to estimate publication bias for OS

## Slide 25
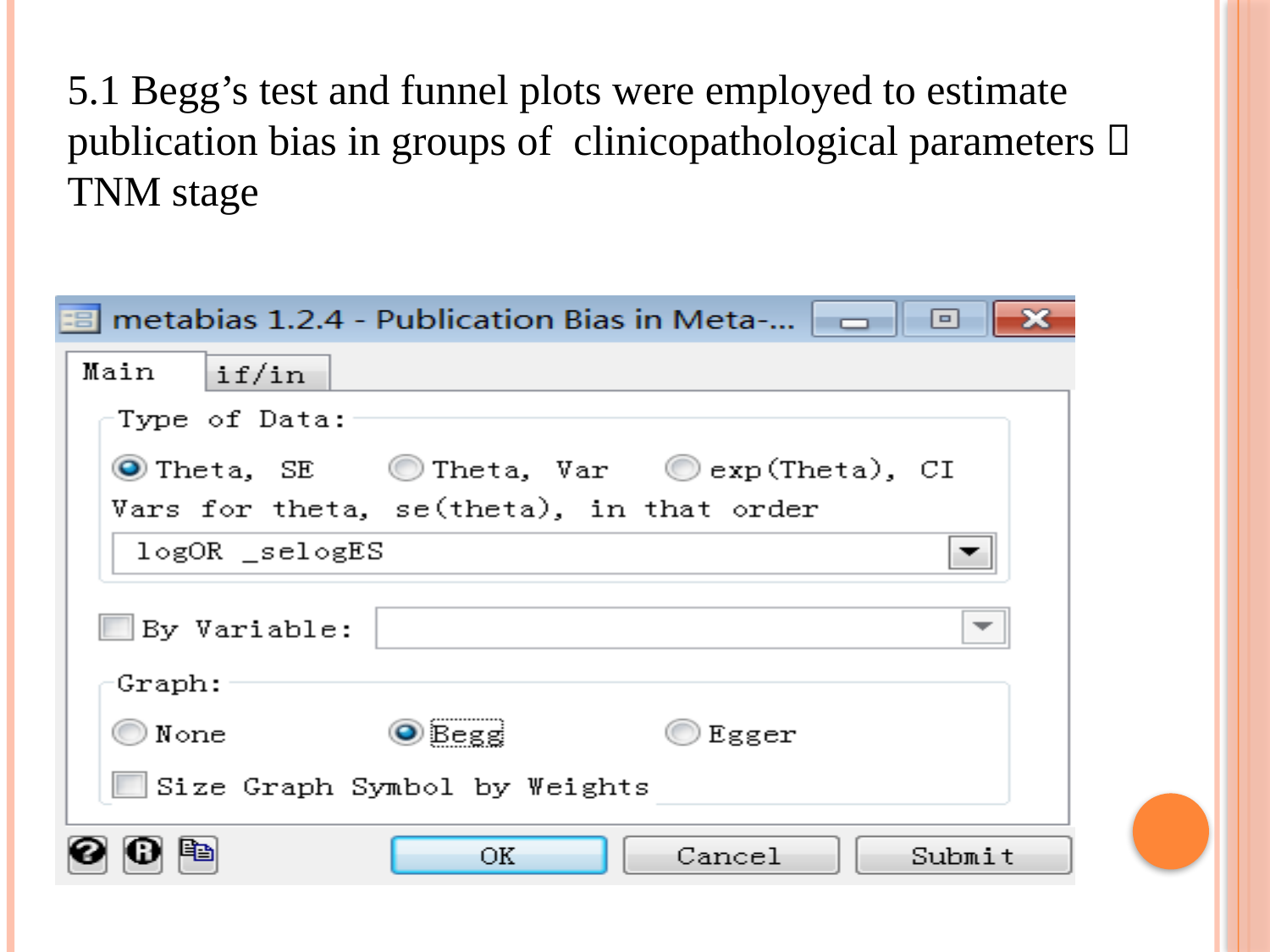

5.1 Begg’s test and funnel plots were employed to estimate publication bias in groups of clinicopathological parameters：
TNM stage

## Slide 26
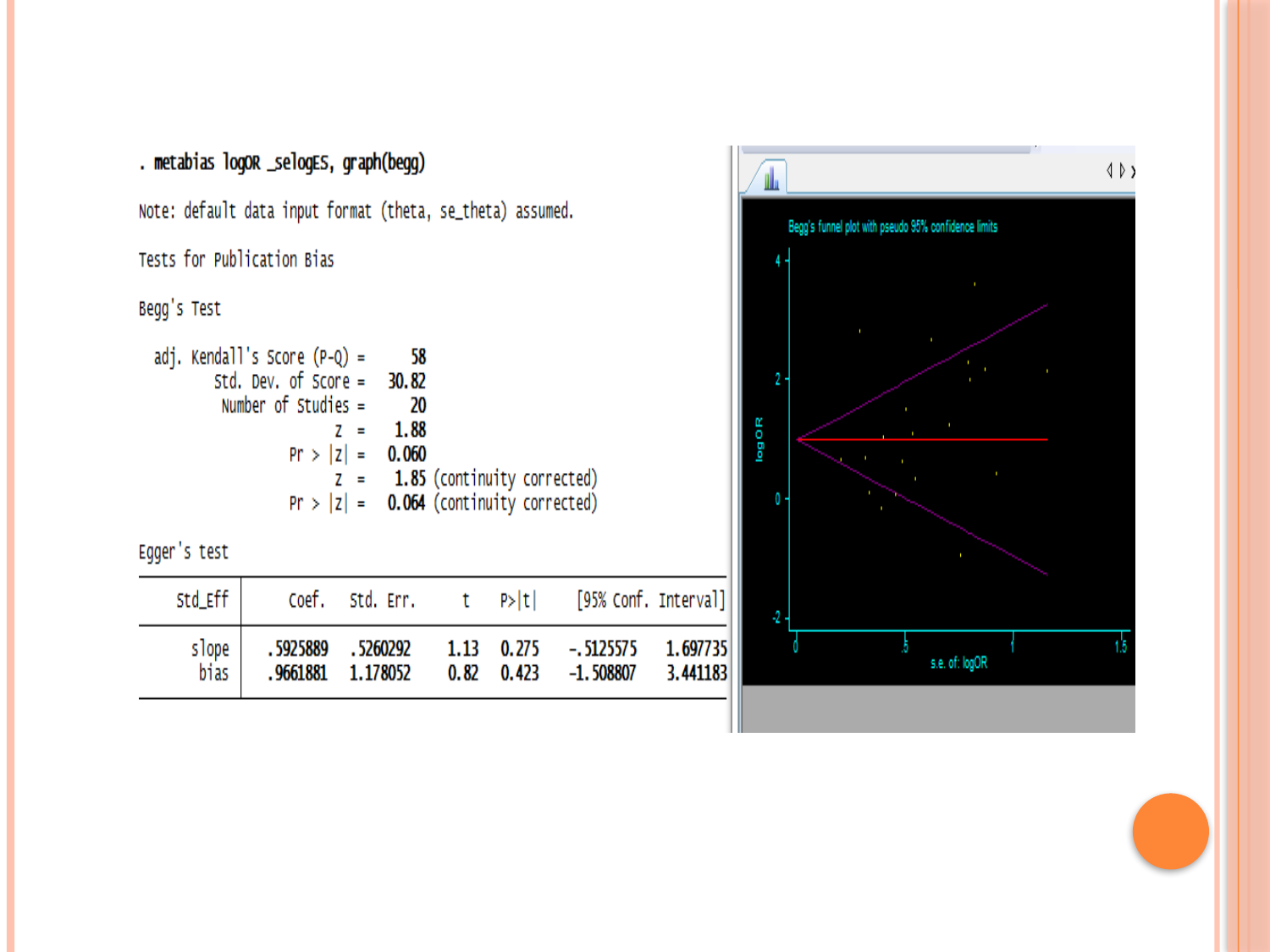

## Slide 27
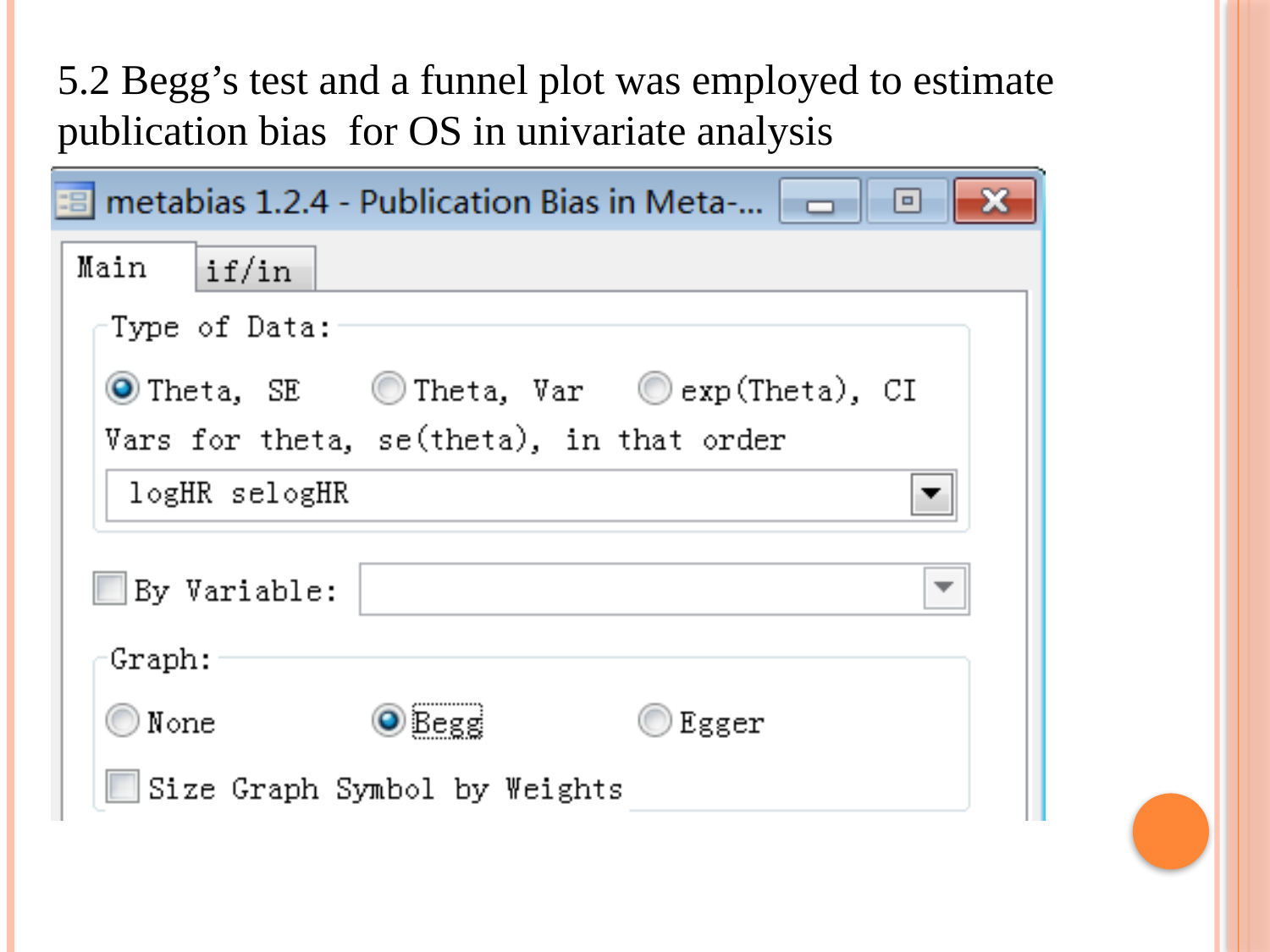

5.2 Begg’s test and a funnel plot was employed to estimate publication bias for OS in univariate analysis

## Slide 28
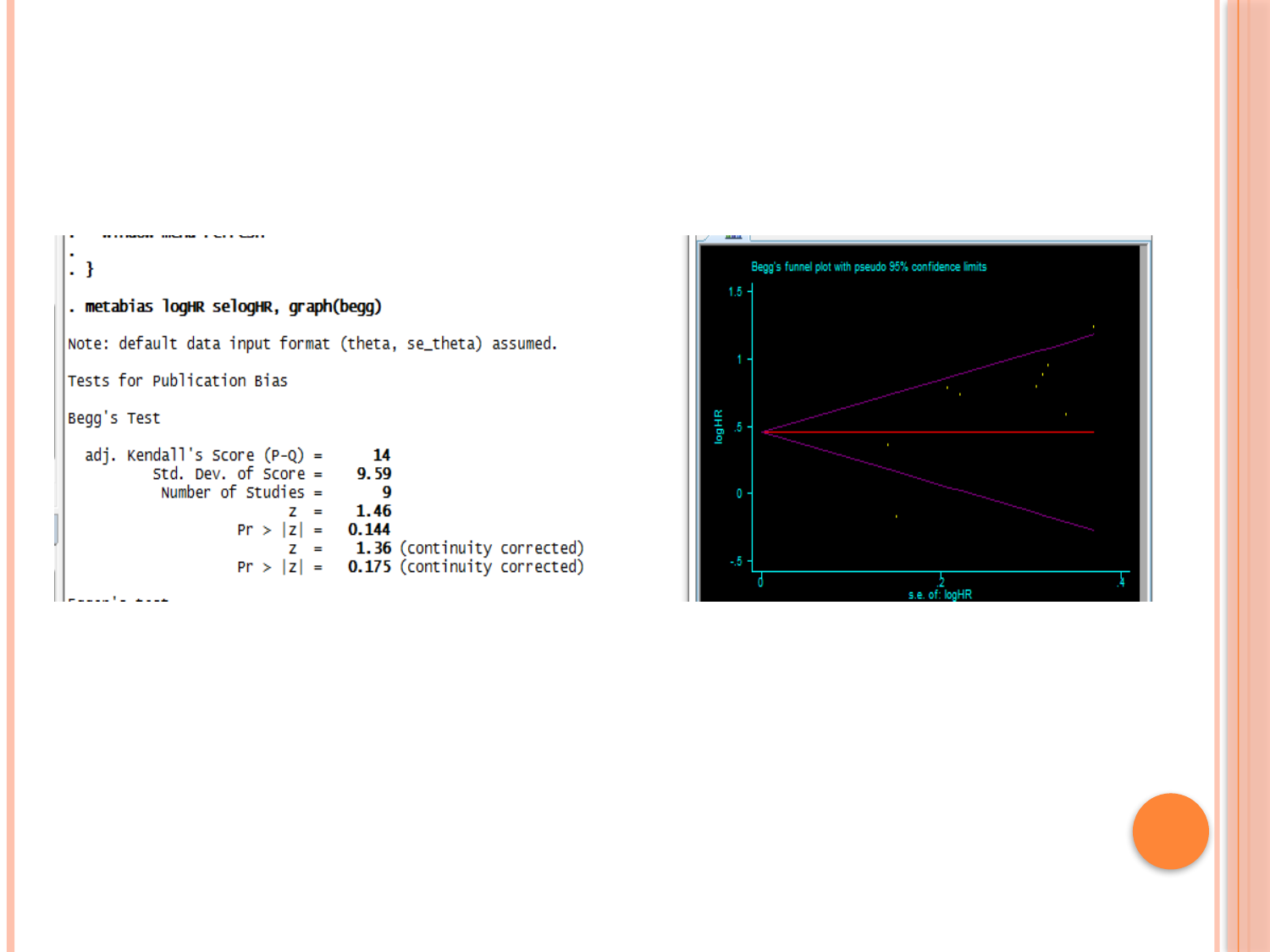

## Slide 29
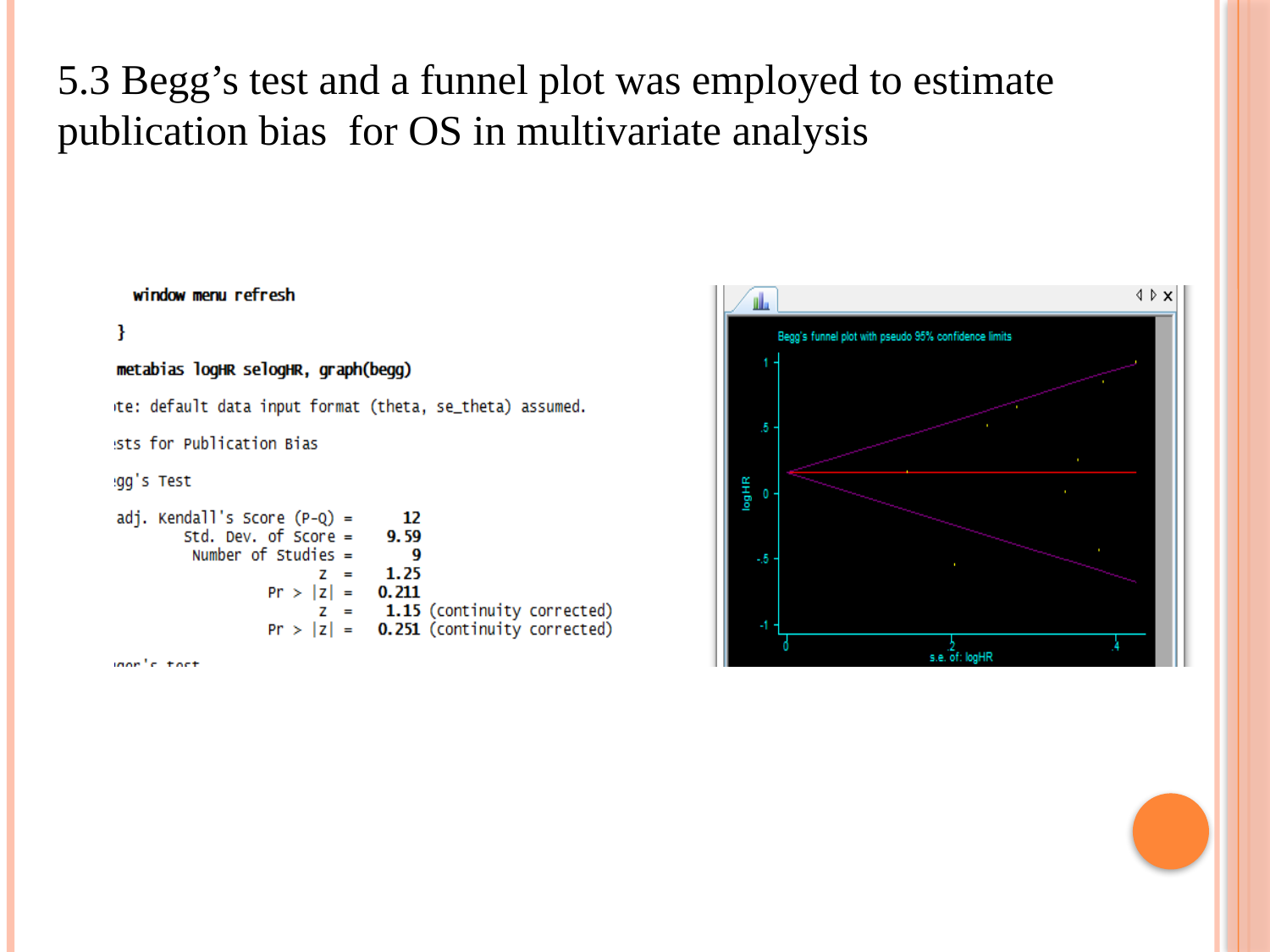

5.3 Begg’s test and a funnel plot was employed to estimate publication bias for OS in multivariate analysis

## Slide 30
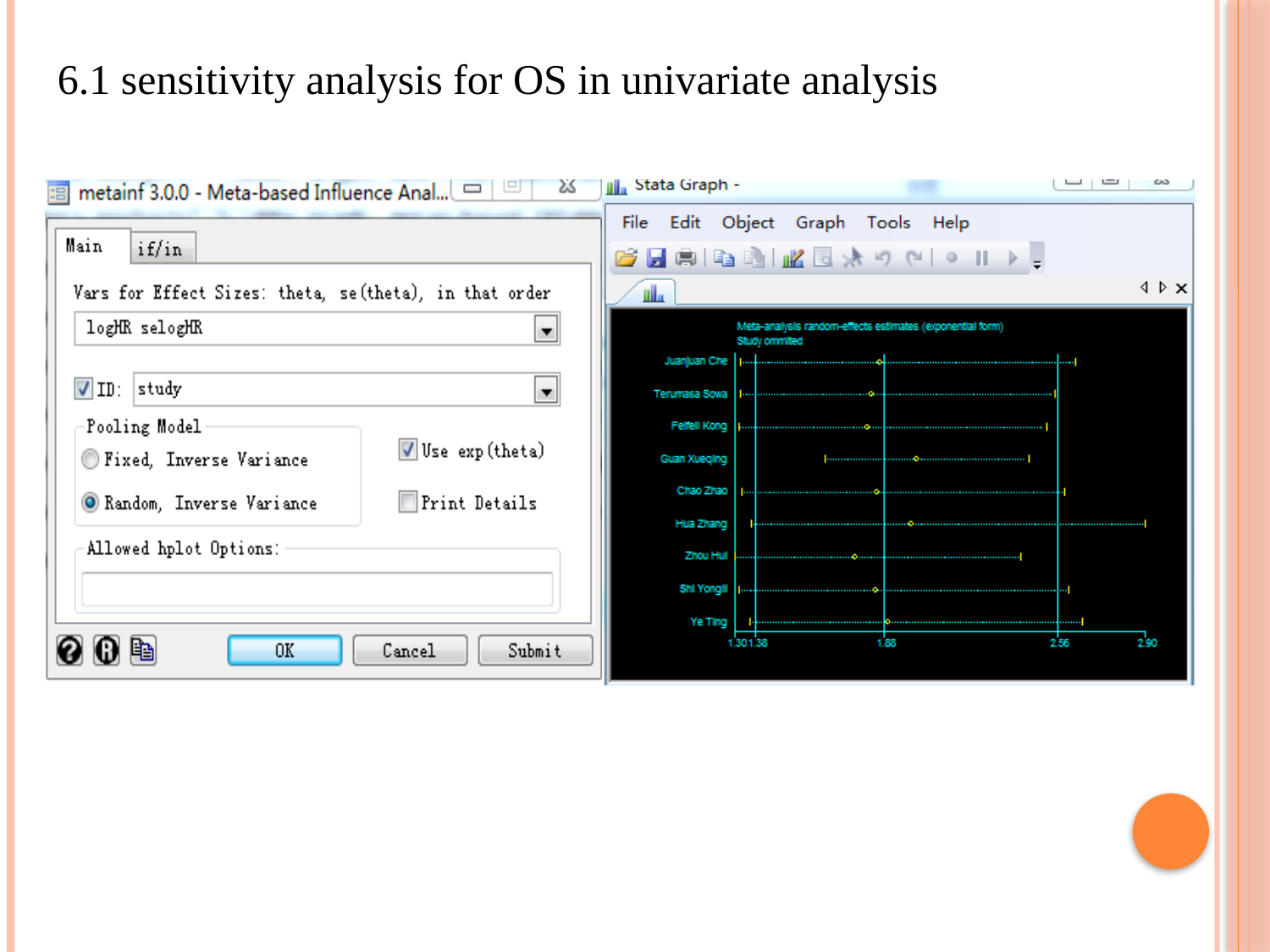

6.1 sensitivity analysis for OS in univariate analysis

## Slide 31
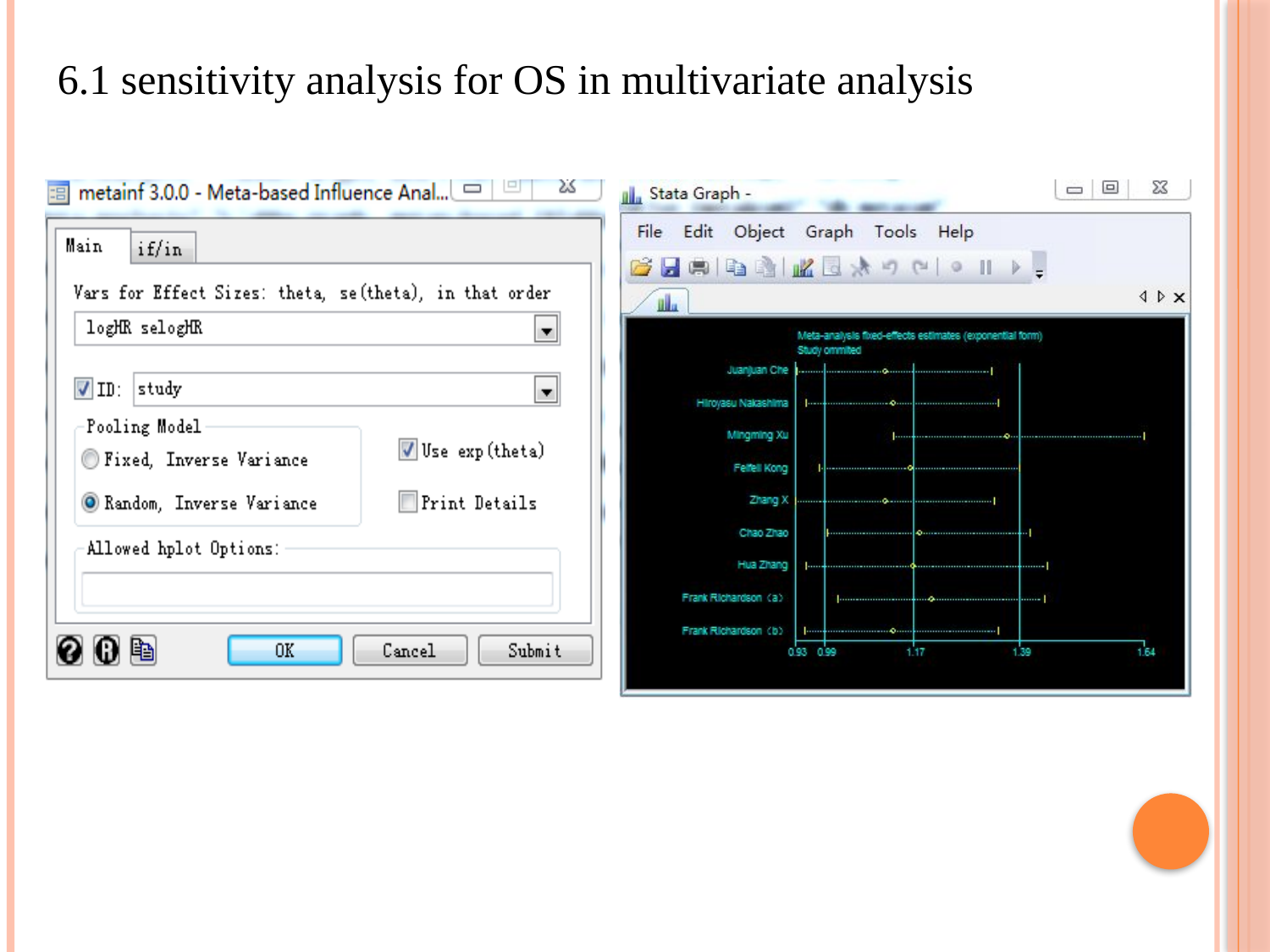

6.1 sensitivity analysis for OS in multivariate analysis

## Slide 32
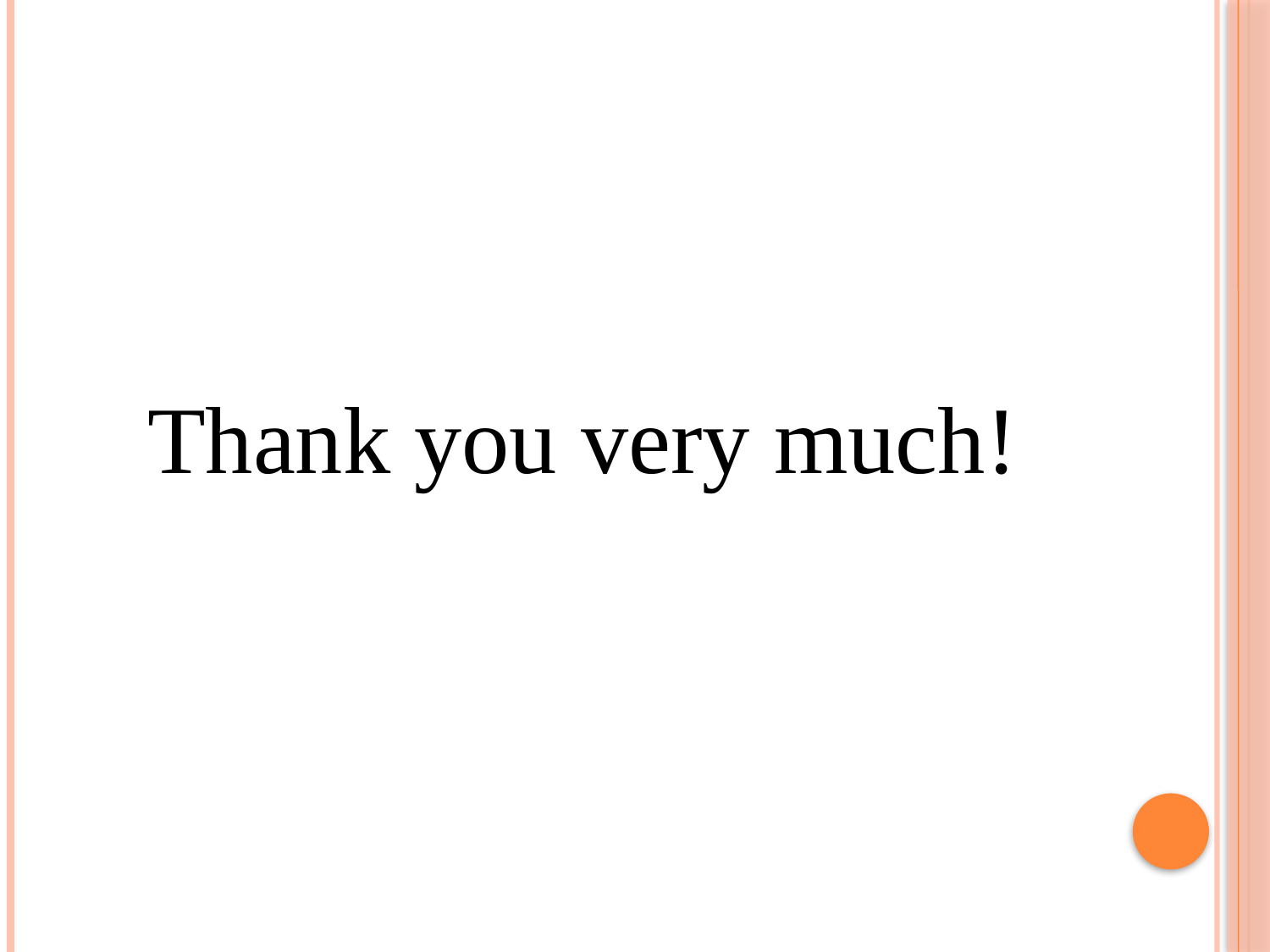

Thank you very much!
