# Supplementary material for: Prognostic Values of Vimentin Expression and Its Clinicopathological Significance in Non-Small Cell Lung Cancer: A Meta-Analysis of Observational Studies with 4118 Cases
Source: PLoS One. 2016 Sep 22;11(9):e0163162. doi: 10.1371/journal.pone.0163162 (PMC5033348; doi:10.1371/journal.pone.0163162)
Supplement: S2 File — (DOCX) [file pone.0163162.s002.docx]

**Studies exclusion**

1. **Studies didn’t provide information for prognosis[**[**1-7**](#_ENREF_1)**].**
2. **Studies had no effective data to estimated HR and its 95% CI[**[**8-13**](#_ENREF_8)**].**
3. **Trails using animals or cell lines or others but not tumor tissues[**[**14-27**](#_ENREF_14)**].**
4. **Trails detected vimentin combined other markers to estimate its clinical significance[**[**28**](#_ENREF_28)**].**
5. **Reviews[**[**29-31**](#_ENREF_29)**].**

**Reference**

1. Upton MP, Hirohashi S, Tome Y, Miyazawa N, Suemasu K, Shimosato Y. Expression of vimentin in surgically resected adenocarcinomas and large cell carcinomas of lung. The American journal of surgical pathology. 1986;10(8):560-7. PubMed PMID: 2426981.

2. Zhang Y, Zhao Y, Jiang G, Zhang X, Zhao H, Wu J, et al. Impact of p120-catenin isoforms 1A and 3A on epithelial mesenchymal transition of lung cancer cells expressing E-cadherin in different subcellular locations. PloS one. 2014;9(2):e88064. doi: 10.1371/journal.pone.0088064. PubMed PMID: 24505377; PubMed Central PMCID: PMC3913724.

3. Kaseda K, Ishii G, Aokage K, Takahashi A, Kuwata T, Hishida T, et al. Identification of intravascular tumor microenvironment features predicting the recurrence of pathological stage I lung adenocarcinoma. Cancer science. 2013;104(9):1262-9. doi: 10.1111/cas.12219. PubMed PMID: 23786153.

4. Morra L, Rechsteiner M, Casagrande S, von Teichman A, Schraml P, Moch H, et al. Characterization of periostin isoform pattern in non-small cell lung cancer. Lung cancer. 2012;76(2):183-90. doi: 10.1016/j.lungcan.2011.10.013. PubMed PMID: 22079858.

5. Pallier K, Cazes A, El Khattabi L, Lecchi C, Desroches M, Danel C, et al. DeltaN TP63 reactivation, epithelial phenotype maintenance, and survival in lung squamous cell carcinoma. Tumour biology : the journal of the International Society for Oncodevelopmental Biology and Medicine. 2012;33(1):41-51. doi: 10.1007/s13277-011-0239-5. PubMed PMID: 21986963.

6. Miura N, Yano T, Shoji F, Kawano D, Takenaka T, Ito K, et al. Clinicopathological significance of Sip1-associated epithelial mesenchymal transition in non-small cell lung cancer progression. Anticancer research. 2009;29(10):4099-106. PubMed PMID: 19846957.

7. Kun C ML, Miao L, Jianhua L. Expression and clinical significant of twist, E-cadherin and vimentin in NSCLC. Journal of liaoning university. 2011;38(3):235-9.

8. Pelletier MP, Edwardes MD, Michel RP, Halwani F, Morin JE. Prognostic markers in resectable non-small cell lung cancer: a multivariate analysis. Canadian journal of surgery Journal canadien de chirurgie. 2001;44(3):180-8. PubMed PMID: 11407827; PubMed Central PMCID: PMC3699112.

9. Soltermann A, Tischler V, Arbogast S, Braun J, Probst-Hensch N, Weder W, et al. Prognostic significance of epithelial-mesenchymal and mesenchymal-epithelial transition protein expression in non-small cell lung cancer. Clinical cancer research : an official journal of the American Association for Cancer Research. 2008;14(22):7430-7. doi: 10.1158/1078-0432.CCR-08-0935. PubMed PMID: 19010860.

10. Feng J, Zhang X, Zhu H, Wang X, Ni S, Huang J. FoxQ1 overexpression influences poor prognosis in non-small cell lung cancer, associates with the phenomenon of EMT. PloS one. 2012;7(6):e39937. doi: 10.1371/journal.pone.0039937. PubMed PMID: 22761930; PubMed Central PMCID: PMC3386178.

11. Hao L, Zhao X, Zhang B, Li C, Wang C. Positive expression of pro-opiomelanocortin (POMC) is a novel independent poor prognostic marker in surgically resected non-small cell lung cancer. Tumour biology : the journal of the International Society for Oncodevelopmental Biology and Medicine. 2015;36(3):1811-7. doi: 10.1007/s13277-014-2784-1. PubMed PMID: 25377161.

12. Hirano H, Maeda H, Takeuchi Y, Susaki Y, Kobayashi R, Hayashi A, et al. Lymphatic invasion of micropapillary cancer cells is associated with a poor prognosis of pathological stage IA lung adenocarcinomas. Oncology letters. 2014;8(3):1107-11. doi: 10.3892/ol.2014.2284. PubMed PMID: 25120667; PubMed Central PMCID: PMC4114577.

13. Yao L, Sun B, Zhao X, Zhao X, Gu Q, Dong X, et al. Overexpression of Wnt5a promotes angiogenesis in NSCLC. BioMed research international. 2014;2014:832562. doi: 10.1155/2014/832562. PubMed PMID: 24999479; PubMed Central PMCID: PMC4066942.

14. Song Y, Zhang C, Cao Z, Xu J, Wang L, Lin X. [Significance of epithelial-mesenchaymal transition phenotype in invasive tumor front cells of lung squamous cell carcinoma]. Zhongguo fei ai za zhi = Chinese journal of lung cancer. 2014;17(4):315-20. doi: 10.3779/j.issn.1009-3419.2014.04.05. PubMed PMID: 24758906.

15. Song A, Kim TM, Kim DW, Kim S, Keam B, Lee SH, et al. Molecular Changes Associated with Acquired Resistance to Crizotinib in ROS1-Rearranged Non-Small Cell Lung Cancer. Clinical cancer research : an official journal of the American Association for Cancer Research. 2015;21(10):2379-87. doi: 10.1158/1078-0432.CCR-14-1350. PubMed PMID: 25688157.

16. Burgess HJ, Kerr ME. Cytokeratin and vimentin co-expression in 21 canine primary pulmonary epithelial neoplasms. Journal of veterinary diagnostic investigation : official publication of the American Association of Veterinary Laboratory Diagnosticians, Inc. 2009;21(6):815-20. PubMed PMID: 19901281.

17. Schliekelman MJ, Taguchi A, Zhu J, Dai X, Rodriguez J, Celiktas M, et al. Molecular portraits of epithelial, mesenchymal, and hybrid States in lung adenocarcinoma and their relevance to survival. Cancer research. 2015;75(9):1789-800. doi: 10.1158/0008-5472.CAN-14-2535. PubMed PMID: 25744723.

18. Zhang X, Miao Y, Yu X, Zhang Y, Jiang G, Liu Y, et al. C6orf106 enhances NSCLC cell invasion by upregulating vimentin, and downregulating E-cadherin and P120ctn. Tumour biology : the journal of the International Society for Oncodevelopmental Biology and Medicine. 2015;36(8):5979-85. doi: 10.1007/s13277-015-3274-9. PubMed PMID: 25736925.

19. Nurwidya F, Takahashi F, Kobayashi I, Murakami A, Kato M, Minakata K, et al. Treatment with insulin-like growth factor 1 receptor inhibitor reverses hypoxia-induced epithelial-mesenchymal transition in non-small cell lung cancer. Biochemical and biophysical research communications. 2014;455(3-4):332-8. doi: 10.1016/j.bbrc.2014.11.014. PubMed PMID: 25446090.

20. Suh SS, Yoo JY, Cui R, Kaur B, Huebner K, Lee TK, et al. FHIT suppresses epithelial-mesenchymal transition (EMT) and metastasis in lung cancer through modulation of microRNAs. PLoS genetics. 2014;10(10):e1004652. doi: 10.1371/journal.pgen.1004652. PubMed PMID: 25340791; PubMed Central PMCID: PMC4207614.

21. Merikallio H, T TT, Paakko P, Makitaro R, Kaarteenaho R, Lehtonen S, et al. Slug is associated with poor survival in squamous cell carcinoma of the lung. International journal of clinical and experimental pathology. 2014;7(9):5846-54. PubMed PMID: 25337226; PubMed Central PMCID: PMC4203197.

22. Zhao Z, Cheng X, Wang Y, Han R, Li L, Xiang T, et al. Metformin inhibits the IL-6-induced epithelial-mesenchymal transition and lung adenocarcinoma growth and metastasis. PloS one. 2014;9(4):e95884. doi: 10.1371/journal.pone.0095884. PubMed PMID: 24789104; PubMed Central PMCID: PMC4005743.

23. Gao L, Liu J, Zhang B, Zhang H, Wang D, Zhang T, et al. Functional MUC4 suppress epithelial-mesenchymal transition in lung adenocarcinoma metastasis. Tumour biology : the journal of the International Society for Oncodevelopmental Biology and Medicine. 2014;35(2):1335-41. doi: 10.1007/s13277-013-1178-0. PubMed PMID: 24037917.

24. Yauch RL, Januario T, Eberhard DA, Cavet G, Zhu W, Fu L, et al. Epithelial versus mesenchymal phenotype determines in vitro sensitivity and predicts clinical activity of erlotinib in lung cancer patients. Clinical cancer research : an official journal of the American Association for Cancer Research. 2005;11(24 Pt 1):8686-98. doi: 10.1158/1078-0432.CCR-05-1492. PubMed PMID: 16361555.

25. Sun M, Liu XH, Wang KM, Nie FQ, Kong R, Yang JS, et al. Downregulation of BRAF activated non-coding RNA is associated with poor prognosis for non-small cell lung cancer and promotes metastasis by affecting epithelial-mesenchymal transition. Molecular cancer. 2014;13:68. doi: 10.1186/1476-4598-13-68. PubMed PMID: 24655544; PubMed Central PMCID: PMC3998010.

26. Mimae T, Okada M, Hagiyama M, Miyata Y, Tsutani Y, Inoue T, et al. Upregulation of notch2 and six1 is associated with progression of early-stage lung adenocarcinoma and a more aggressive phenotype at advanced stages. Clinical cancer research : an official journal of the American Association for Cancer Research. 2012;18(4):945-55. doi: 10.1158/1078-0432.CCR-11-1946. PubMed PMID: 22190591.

27. Tischler V, Pfeifer M, Hausladen S, Schirmer U, Bonde AK, Kristiansen G, et al. L1CAM protein expression is associated with poor prognosis in non-small cell lung cancer. Molecular cancer. 2011;10:127. doi: 10.1186/1476-4598-10-127. PubMed PMID: 21985405; PubMed Central PMCID: PMC3198986.

28. Ren S, Su C, Wang Z, Li J, Fan L, Li B, et al. Epithelial phenotype as a predictive marker for response to EGFR-TKIs in non-small cell lung cancer patients with wild-type EGFR. International journal of cancer. 2014;135(12):2962-71. doi: 10.1002/ijc.28925. PubMed PMID: 24771540.

29. Kidd ME, Shumaker DK, Ridge KM. The role of vimentin intermediate filaments in the progression of lung cancer. American journal of respiratory cell and molecular biology. 2014;50(1):1-6. doi: 10.1165/rcmb.2013-0314TR. PubMed PMID: 23980547; PubMed Central PMCID: PMC3930939.

30. Yi H, Ku NO. Intermediate filaments of the lung. Histochemistry and cell biology. 2013;140(1):65-9. doi: 10.1007/s00418-013-1105-x. PubMed PMID: 23765163.

31. Satelli A, Li S. Vimentin in cancer and its potential as a molecular target for cancer therapy. Cellular and molecular life sciences : CMLS. 2011;68(18):3033-46. doi: 10.1007/s00018-011-0735-1. PubMed PMID: 21637948; PubMed Central PMCID: PMC3162105.
