# Supplementary material for: Prognostic Values of Vimentin Expression and Its Clinicopathological Significance in Non-Small Cell Lung Cancer: A Meta-Analysis of Observational Studies with 4118 Cases
Source: PLoS One. 2016 Sep 22;11(9):e0163162. doi: 10.1371/journal.pone.0163162 (PMC5033348; doi:10.1371/journal.pone.0163162)
Supplement: S3 File — (DOC) [file pone.0163162.s003.doc]

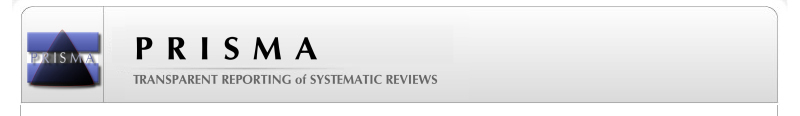
**PRISMA 2009 Flow Diagram**

**Screening**

**Included**

**Eligibility**

**Identification**

Records identified through database searching
(n =7171)

Additional records identified through other sources
(n = 0)

Records after duplicates removed
(n =5515)

Records screened
(n = 1656)

Records excluded
(n =1593)

Full-text articles assessed for eligibility
(n = 63)

Full-text articles excluded, with reasons
(n =31)

Studies included in qualitative synthesis
(n = 32)

Studies included in quantitative synthesis (meta-analysis)
(n = 32)
